# Improved Wheat Growth and Yield by Delayed Leaf Senescence Using Developmentally Regulated Expression of a Cytokinin Biosynthesis Gene

**DOI:** 10.3389/fpls.2019.01285

**Published:** 2019-10-18

**Authors:** Sameer Joshi, Anil Choukimath, Daniel Isenegger, Joe Panozzo, German Spangenberg, Surya Kant

**Affiliations:** ^1^Agriculture Victoria, Grains Innovation Park, Horsham, VIC, Australia; ^2^Agriculture Victoria, AgriBio, Centre for AgriBioscience, Bundoora, VIC, Australia; ^3^School of Applied Systems Biology, La Trobe University, Bundoora, VIC, Australia; ^4^Centre for Agricultural Innovation, The University of Melbourne, VIC, Australia

**Keywords:** cytokinin, leaf water potential, normalized difference vegetation index, stress susceptibility index, transgenic

## Abstract

Delaying leaf senescence in plants, especially under water stress conditions, can help to maintain the remobilization of stored nutrients in source–sink relationships, thus leading to improved crop yields. Leaf senescence can be delayed by plant hormones such as cytokinin. Here, the *Isopentenyl transferase (IPT)* gene, encoding a cytokinin biosynthesis enzyme, driven by a modified *AtMYB32xs* promoter was transformed into wheat. Transgenic wheat plants exhibited delayed leaf senescence, retaining chlorophyll for longer under controlled environment conditions. Selected independent transgenic events and their corresponding nulls were grown under field conditions for two consecutive years under well-watered and water stress treatments using automated rainout shelters. Three independent transgenic events had improved canopy green cover, lower canopy temperatures, and higher leaf water potential than their respective non-transgenic nulls, with no abnormality in morphology and phenology. Increased grain yield was observed in transgenic events under both water treatments, with the yield increase more pronounced under water stress (26–42%). These results have shown that delayed leaf senescence using the chimeric transgene *AtMYB32xs-p::IPT* can be a useful strategy to achieve grain yield gains in wheat and potentially other crops for sustainable food production.

## Introduction

Leaf senescence is a highly regulated form of programmed cell death accompanied by protein and nucleic acid degradation, and chloroplast disintegration (Gan and Amasino, 1995; Simeonova et al., 2000). Delayed leaf senescence permits source tissue to continue producing, recycling, and remobilizing photosynthates for extended periods, ultimately contributing to enhanced grain yield and quality (Gregersen et al., 2008). Early or untimely leaf senescence results in the inadequate mobilization of leaf or stem metabolites to reproductive parts i.e. spikes and grains in the case of wheat. The reduced mobilization of metabolites to growing seeds lower grain yield as well as nutritional quality (Gregersen et al., 2013). Senescence accelerates in the presence of biotic or abiotic stresses. Water stress, for example, affects the plant growth and development during vegetative and reproductive phases. The effect is more pronounced at the reproductive phase and untimely senescence shown to affect drought tolerance (Rivero et al., 2007; Gregersen et al., 2008; Gregersen et al., 2013).

Cytokinins are a class of plant hormones vital for promoting cell division, growth, and differentiation and therefore influences developmental and physiological processes including germination, flowering, seed development, and leaf senescence (Miller et al., 1956; Gan and Amasino, 1996; Haberer and Kieber, 2002). Cytokinins have been shown to play a role in delaying leaf senescence in several plant species (Akiyoshi et al., 1984; Smigocki and Owens, 1988; Robson et al., 2004; Rivero et al., 2007; Sýkorová et al., 2008; Peleg et al., 2011; Qin et al., 2011). In these studies, the *Agrobacterium tumefaciens Isopentenyl transferase (IPT)* gene, a key enzyme which catalyzes a critical step in cytokinin biosynthesis, was used to increase the level of endogenous cytokinin. However, the correct regulation of the *IPT* gene is necessary to gain the crucial benefits of increased cytokinin levels, without deleterious changes to morphology and phenology. To date, several stress and developmentally induced promoters have been used to control the *IPT* gene in species such as tobacco and wheat, with varying degrees of success. These have included the heat inducible promoter *HSP70*, the leaf senescence induced *SAG12* promoter, and the stress and maturation induced *SARK* promoter (Medford et al., 1989; Gan and Amasino, 1995; Rivero et al., 2007; Sýkorová et al., 2008). The *SAG12* promoter controlling *IPT* showed several benefits including delayed chlorophyll degradation and increased nitrogen fluxes, although plants also showed abnormal sink-to-source relationships affecting grain yield (Jordi et al., 2000; Sýkorová et al., 2008; Cowan et al., 2005). Transgenic plants where *IPT* expression was regulated by the *SARK* promoter maintained higher water content and photorespiration rates, as well as showing delayed leaf senescence, drought tolerance, and increased yield and quality, compared to wild type plants, under water stress conditions only (Rivero et al., 2007; Peleg et al., 2011; Qin et al., 2011; Kuppu et al., 2013).

The use of the *IPT* gene in delaying leaf senescence under different abiotic stresses has been explored using constitutive, stress associated, or senescence inducible promoters. However, the advantages gained for yield increase may have not been fully explored under a non-stress condition, such as well watered and/or optimum nutrient levels. The spatio-temporal activity of the promoter plays a critical role in the regulation of the *IPT* gene in delaying leaf senescence, and when exploring the benefits of this gene under stressed and non-stressed conditions. Therefore, it may be desirable to use a promoter that can developmentally regulate *IPT* expression, rather than just during the onset of processes such as senescence and maturation. To overcome these issues and appropriately regulate the *IPT* gene, the *Arabidopsis thaliana MYB32* (*AtMYB32*) promoter, which can activate gene expression during the development of lateral roots and anthers in *A. thaliana* (Lin et al., 2003; Preston et al., 2004), was used. The *AtMYB32* promoter in this study was modified, where the root specific expression motif was removed, and promoter named as *AtMYB32xs*. The details of *AtMYB32xs* promoter are provided in the *Materials and Methods* section. The modified *AtMYB32xs* promoter still consisted of the anther specific motif which was adequate to express the *IPT* gene during flowering, as well as during senescence. Our previous study in canola showed that transgenic plants expressing *IPT* under a modified *AtMYB32xs* promoter exhibited delayed leaf senescence as well as higher yield compared to wild type plants (Kant et al., 2015). Following on from these results in the dicot canola, the role of *IPT* and delayed leaf senescence in the monocot wheat was of interest under both controlled environment and field conditions. To achieve this, custom designed rain sensor automated and fully portable rainout shelters were used to impose precise water stress treatments in the field (Kant et al., 2017). In this study, the integration of the chimeric transgene construct *AtMYB32xs-p::IPT* in wheat was shown to delay leaf senescence and have a beneficial effect on the yield and yield related traits under both well-watered and water stress conditions.

## Materials and Methods

### Plant Transformation and Molecular Analysis of Transformants

Transgenic wheat plants expressing the *IPT* gene under the control of transcription factor gene promoter *AtMYB32* (AT4G34990) were generated by *Agrobacterium tumefaciens* mediated transformation into the bread wheat (*Triticum aestivum* L.) variety Bobwhite-26. The *AtMYB32* promoter (942 bp in length) contains root specific, pollen, and MYB related motifs. The root specific motif was flanked by *XcmI* and *SspI* restriction sites. The respective restriction enzymes were used to cut out the root specific motif sequence to avoid root specific expression of *IPT* gene and formation of any root abnormalities. This modified promoter was named as *AtMYB32xs* (**Supplementary Figure 1**). The transgene sequence (*AtMYB32xs-p::IPT::CaMV35s-t*) was synthesized using the Invitrogen™ GeneArt™ Gene Synthesis service (Thermo Fisher Scientific, USA) and was then transferred into the T-DNA region of a binary expression vector using GATEWAY*^®^* recombination cloning technology (Invitrogen, USA) in a Gateway-enabled pPZP binary vector (Hajdukiewicz et al., 1994). The T-DNA region of the binary vector also contained a wheat codon optimized selectable marker cassette (*OsActin-p::bar::CaMV35s-t*) that comprised a synthetically constructed *BAR* gene (Thompson et al., 1987) that encoded for the *Phosphinothricin acetyltransferase* (*PAT*) gene, and regulatory sequences from the rice actin promoter and Cauliflower Mosaic Virus terminator (**Supplementary Figure 2**).

The T_1_ transgenic wheat plants were obtained from primary transformation events (T_0_) by self-pollination. The selected homozygous transformants of the T_2_ generation were advanced to T_4_ generation to undertake controlled environment and field evaluation. Sister non-transgenic events, referred to here on as nulls, which underwent the same transformation process but were confirmed as non-transgenic, were also propagated and used as comparative controls in this study. The putative independent transformation events were analyzed for the transgene copy number and the transcriptional competence of the gene construct was verified on T_1_ plants from selected events using reverse transcriptase-PCR (**Supplementary Table 1** and **Supplementary Figure 3**).

### Detached Leaf Assay

To assess transgenic events for delayed leaf senescence, a detached leaf assay was performed on plants grown in a controlled environment (24–15°C and a 14 h/10 h light/dark photoperiod). Transgenic and nulls plants from three independent events were grown for 75 days, after which fully opened second-from-the-top leaves were harvested when plants were at booting stage (Zadoks (Z) 45) (Zadoks et al., 1974), and placed on the moist filter paper in Petri dishes. The leaf sheath was covered with moist filter paper for 16 days. At the end, digital photographs were taken, and the chlorophyll level was measured using a SPAD chlorophyll meter (SPAD-502 Plus; Konika Minolta, Spectrum Technologies Inc., USA).

### Field Experiments

The field experiments were conducted under the license “DIR 122—limited and controlled release of wheat genetically modified for enhanced yield stability” issued by the Office of the Gene Technology Regulator, Australia. The field experiments were conducted for two consecutive years in 2014 (36°43′53.800″S 142°06′03.000″E) and in 2015 (36°43′59.052″S 142°06′3.096″E) at the Plant Breeding Centre, Horsham, Victoria, Australia under well-watered and water stress treatments. Horsham is in the medium rainfall zone with a temperate climate, vertisol soils with cracking clays. In the 2014 field experiment, a total of 13 genotypes were planted that included six transgenic events, named 31, 32, 33, 35, 37, and 38, and their respective nulls, and Bobwhite-26 (wild type). The best performing genotypes from the 2014 experimental results were selected for the 2015 field experiment, resulting in 11 genotypes being planted, including five transgenic events, 31, 33, 35, 37, and 38, and their respective nulls, and Bobwhite-26 (wild type). The experiments for both years were conducted in a split plot design with watering treatments as main plots and genotypes as sub-plots with three replications. For each plot (4 m × 1 m), seed was sown at a rate of 135 plants/m^2^ in six rows at 15 cm apart. Both experiments were conducted during the winter growing season, from May to December. The soil moisture at depths of 100, 200, 600, and 1000 mm below soil surface was measured using a soil profile probe (PR2/6; Delta-T devices, UK) every week from mid tillering until harvest. Access tubes of 1.2 m in length, in which the probe was inserted for measurements, were installed in the ground at multiple locations within the experimental field.

The plants under the well-watered treatment were irrigated using a conventional drip irrigation system. The plants designated for the water stress treatment were exposed to rain events only at early growth stages allowing for optimal plant establishment. Rain events were excluded on these plants to induce water stress during anthesis and reproductive growth stages using a custom built, portable rainout shelter (Kant et al., 2017). Three rainout shelters were used, each 20 m × 10 m in dimension, equipped with an automated solar powered rain sensor that enabled rainout shelters to cover the plants during rain events to induce the water stress treatment in field conditions (**Supplementary Figure 5**). The rainout shelters were mounted on plastic road barriers filled with water, to allow for portability and were installed with a drainage system to avoid the spillage of rainwater into the experimental plots. Rainfall received during the growing season is detailed in **Supplementary Table 2**.

### Plant Growth, Yield, and Quality Observations

#### Canopy Green Coverage

The normalized difference vegetation index (NDVI) was used as a surrogate measure of canopy green coverage and was recorded at weekly intervals from mid tillering (Z25) until the onset of physiological maturity (Z90). The Crop Circle (ACS 470; Holland Scientific Inc, USA), a reflectance-based instrument, was used to estimate NDVI by scanning over experimental plots and measuring the reflectance at 670 nm (visible spectrum) and 760 nm (near infra-red spectrum). The NDVI values for each plot were calculated using the formula (*R*_760_−*R*_670_)/(*R*_760_+*R*_670_) (Gamon et al., 1995).

#### Canopy Temperature

An infra-red thermometer (Everest 6210L; AGRI Therm III, Everest, USA) was used to measure canopy temperature around solar noon on sunny days every alternate week from mid tillering (Z25) to physiological maturity (Z90). The infra-red thermometer was held 2 m away from the crop canopy at a 45° angle; five temperature measurements were recorded per plot.

#### Leaf Water Potential

The leaf water potential was measured using leaf patch clamp pressure (LPCP) probes (YARA ZIM Plant Technology GmbH, Hennigsdorf, Germany). The measuring principle of LPCP probes has been described previously (Zimmermann et al., 2008; Zimmermann et al., 2013). These probes measure the real-time water status of plants; therefore, the probes can take into account the spatial and temporal dynamics of water reallocation, water loss, and the regulation of stomata aperture. In brief, a representative leaf is clamped between the two magnets of the probe, one of which contains the sensor. These two magnets exert clamp pressure (P_c_) onto the leaf. The leaf water potential is inversely proportional to the magnetic patch pressure (P_P_) (Kant et al., 2014) and is calculated using the following equation:

$$\mathrm{Pp}\;=\;{\left(\frac{b}{\mathrm{aPc}\;+b}\right)}^{\frac{1}{a}}\cdot F_{a}\cdot P_{\mathrm{clamp}\cdot }$$

where a and b are leaf-specific elastic constants equal or larger than unity; F_a_ is dimensionless and is a leaf-specific attenuation factor, which considers that part of P_clamp_ explained by the compression of air-filled spaces and structural elements such as cuticles and cell walls. The F_a_ is practically constant even at very low turgor pressures (ca. 50 kPa) and over long measuring periods (Zimmermann et al., 2008; Westhoff et al., 2009; Fernández et al., 2011). This means P_p_ is small when P_c_ is high and *vice versa*. The temperature (T) and relative humidity (RH) were recorded along with the P_p_. Three replicates of LPCP probes were used for each genotype and watering treatment. During clamping, the leaves were cleaned to remove dust and any foreign objects. The LPCP probes were clamped on the flag leaf of wheat plants at 50% heading (Z55) and removed at physiological maturity (Z90). The clamping procedure was similar to that described in detail by Bramley et al. (2013). The LPCP probes were supported by iron rods to avoid plant injury caused by the magnet dragging down the leaf. The P_p_ readings were recorded every minute. The transgenic and null genotypes were compared for time (minutes) to reach peak P_p_ and relaxation time, for peak P_p_ it was the number of minutes after sunrise to reach the peak value, and relaxation time was the number of minutes after sunset to reach 66% of the lowest value.

#### Phenology

The timing of 50% heading was recorded as days after sowing (DAS) for 50% main stems in each plot to reach heading (Z55) and physiological maturity was recorded as DAS when 90% of plants per plot reached maturity (Z90).

#### Grain Yield and Yield Related Components

The above ground biomass of each plot was hand-harvested from a 1 m^2^ area excluding the plants from outer rows and the edges of each plot. During post-harvest processing, the spikes were threshed in a multi head thresher (Kimseed Multi-thresher CW09; WA, Australia) and grain yield was expressed as tons per hectare.

#### Stress Susceptibility Index

The grain yield obtained under both water stress and well-watered treatments was used to calculate the stress susceptibility index (SSI) of the transgenic and null genotypes using the formula SSI = [1−(Y_t_)/(Y_c_)]/[1−(Ȳ_t_)/(Ȳ_c_)] (Fischer and Maurer, 1978), where Y_t_ and Y_c_ are the yield of the genotype evaluated under water stress and well-watered treatments, respectively, and Ȳ_t_ and Ȳ_c_ are the mean yield of all the transgenic and null genotypes evaluated under water stress and well-watered treatments, respectively.

#### Grain Quality

Grain quality parameters: protein content (%), grain hardness (%), test weight (g), dough stability (BU), and dough extensibility (cm) were measured using near infra-red spectroscopy (Black and Panozzo, 1999).

#### Statistical Analysis

Data was analyzed using GenStat statistical software (VSN International, Ltd, UK), with grain quality data analyzed by ANOVA. The graphs for LPCP probes were prepared using the software Origin (OriginLab, USA). To understand how transgenic events maintained yield under water stress in relation to yield obtained under well-watered conditions, *K*-means cluster analysis was performed using well-watered grain yield (t/ha) and the SSI calculated, using R software (http://www.R-project.org) following the method described in Yu et al. (2017).

## Results

### Selection of Homozygous Lines and Detached Leaf Assay

A differential expression of *IPT* gene was observed among the wheat transformants. The transgenic plants, expressing the *IPT* gene (**Supplementary Figure 3**), were selected for homozygous and single-locus integrations from T_0_ to T_2_ generations using PCR-based analysis for the selectable marker (data not shown). Selected PCR-positive, transgenic homozygous genotypes and their respective nulls were grown in a controlled environment. Higher seed yield and delayed leaf senescence was observed for three transgenic events (33, 35, and 37) compared to their corresponding nulls and the wild type (data not shown).

**Figure 1:**
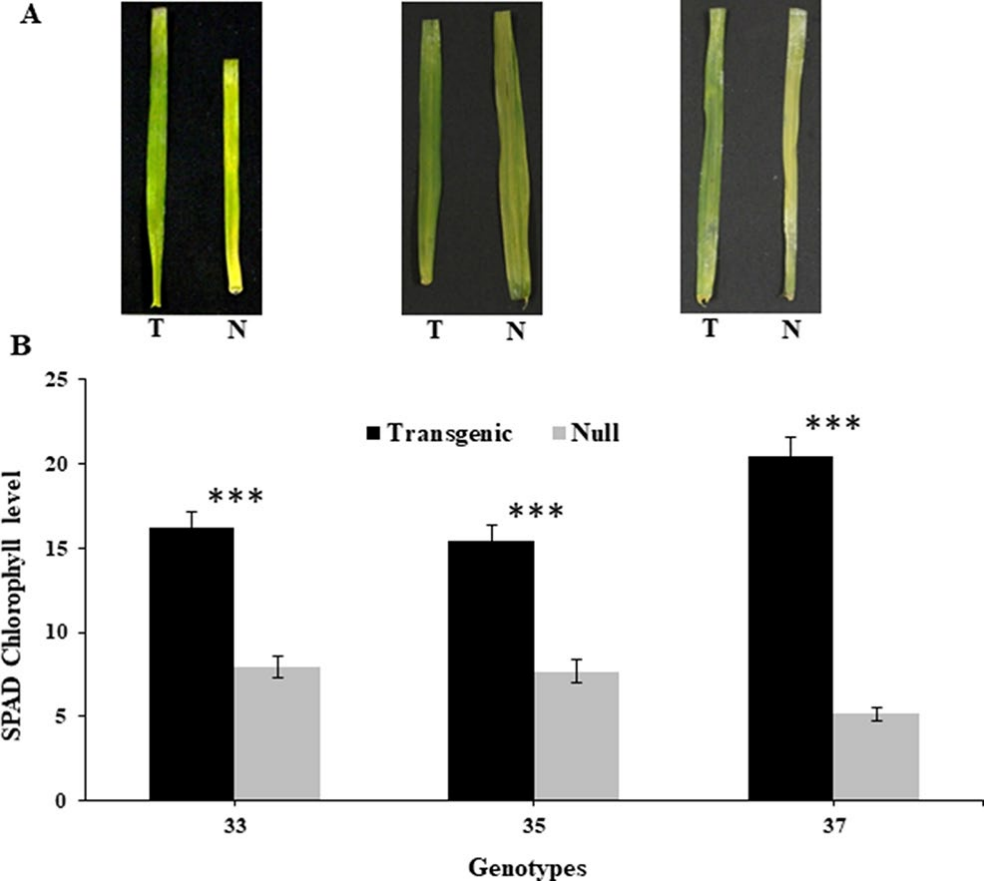
Leaf senescence in transgenic and null lines of wheat from detached leaf assay. Images of representative transgenic and null lines from three independent events taken 16 days after harvest from early booting plants **(A)**, and SPAD chlorophyll levels of the detached leaves from the same three transgenic and null lines taken 16 days after harvest **(B)**. Data represented as mean ± SEM. T, transgenic; N, null. ***Significant differences at P < 0.001.

At the reproductive stage (Z45), harvested leaves on moist filter paper demonstrated that the transgenic leaves remained green for a longer duration than their respective nulls (**Figure 1A**). This was consistent with reduced chlorophyll degradation seen by the measured chlorophyll levels which were higher in leaves of transgenic plants compared to nulls (**Figure 1B**).

### Growth and Phenology of Wheat Plants Under Field Conditions

To address the unpredictability of rainfall and to induce a reliable and precise water stress treatment in field experiments, custom built portable rainout shelters were used to ensure plants were under water stress at crucial reproductive phases. The water deficit stress experienced by the plants was consistent with the soil moisture measurements. Soil moisture was measured over the three growth phases, with soil under well-watered conditions maintained at a higher moisture content than water-stressed soil at almost all depths (**Supplementary Figure 4**). Well-watered soil had more moisture available during the reproductive stages, anthesis, and grain filling, which ensured adequate moisture supply throughout the soil profile. Well-watered plants received less than the long-term average rainfall in both years (**Supplementary Table 2**), and therefore, soil moisture was supplemented by drip irrigation to above long-term average rainfall.

In both experiments, for the number of days to heading and maturity, there was no significant difference between transgenic and null genotypes within the water stress or well-watered treatments (**Table 1**). For the 2014 experiment, the plants reached heading at a similar time under water stress and well-watered treatments. However, in 2015 plants under the water stress treatment headed earlier when compared to well-watered plants. The physiological maturity of plants was found to be earlier in the water stress treatment compared to the well-watered treatment by 19 and 6 days in 2014 and 2015, respectively (**Table 1**). The water stress treatment induced by the rainout shelter therefore shortened the plant growth cycle compared to the well-watered treatment (**Table 1**).

**Table 1:** Phenology of wheat genotypes during field experiments.

	Heading (DAS)				Maturity (DAS)			
	Well-watered		Water stress		Well-watered		Water stress	
	2014	2015	2014	2015	2014	2015	2014	2015
**31T**	125	138	126	132	178	184	159	178
**31N**	125	138	125	133	178	183	157	179
**32T**	126	–	126	–	177	–	160	–
**32N**	126	–	126	–	177	–	160	–
**33T**	126	140	125	132	178	184	159	178
**33N**	125	138	125	133	178	183	159	178
**35T**	125	138	126	132	178	184	160	177
**35N**	124	138	125	132	177	184	157	176
**37T**	126	139	126	132	178	184	160	178
**37N**	126	141	125	139	178	186	159	178
**38T**	126	139	126	133	177	184	159	178
**38N**	126	138	125	133	178	184	159	178
**Mean**	125	139	125	133	178	184	159	178

DAS, days after sowing; T, transgenic; N, null; –, not included in 2015.

### Transgenic Plants Exhibited Enhanced Canopy Green Cover and Lower Canopy Temperature

**Figure 2:**
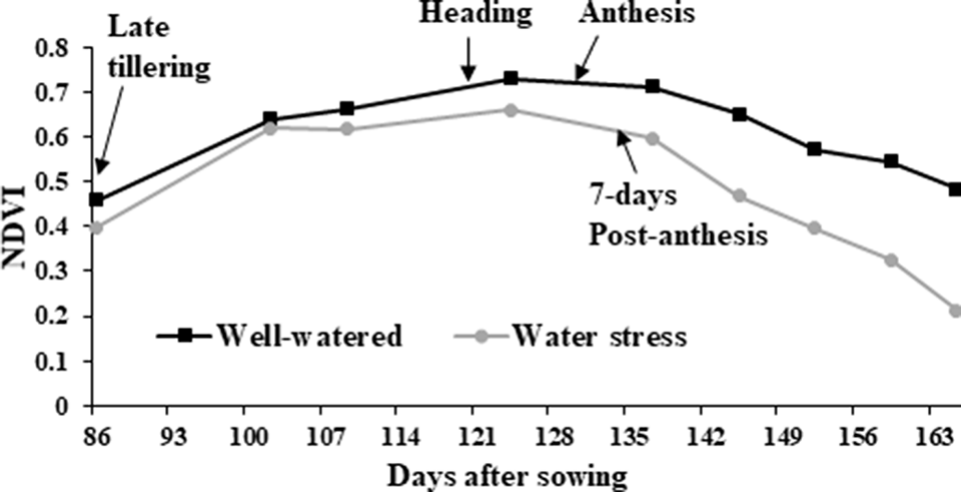
Normalized difference vegetation index (NDVI) in wheat genotypes. Average of all wheat genotypes grown in the 2014 field experiment under well-watered (black line with squares) and water stress (gray line with circles) treatments. NDVI was calculated from late tillering until late reproductive stage using Crop Circle.

**Figure 3:**
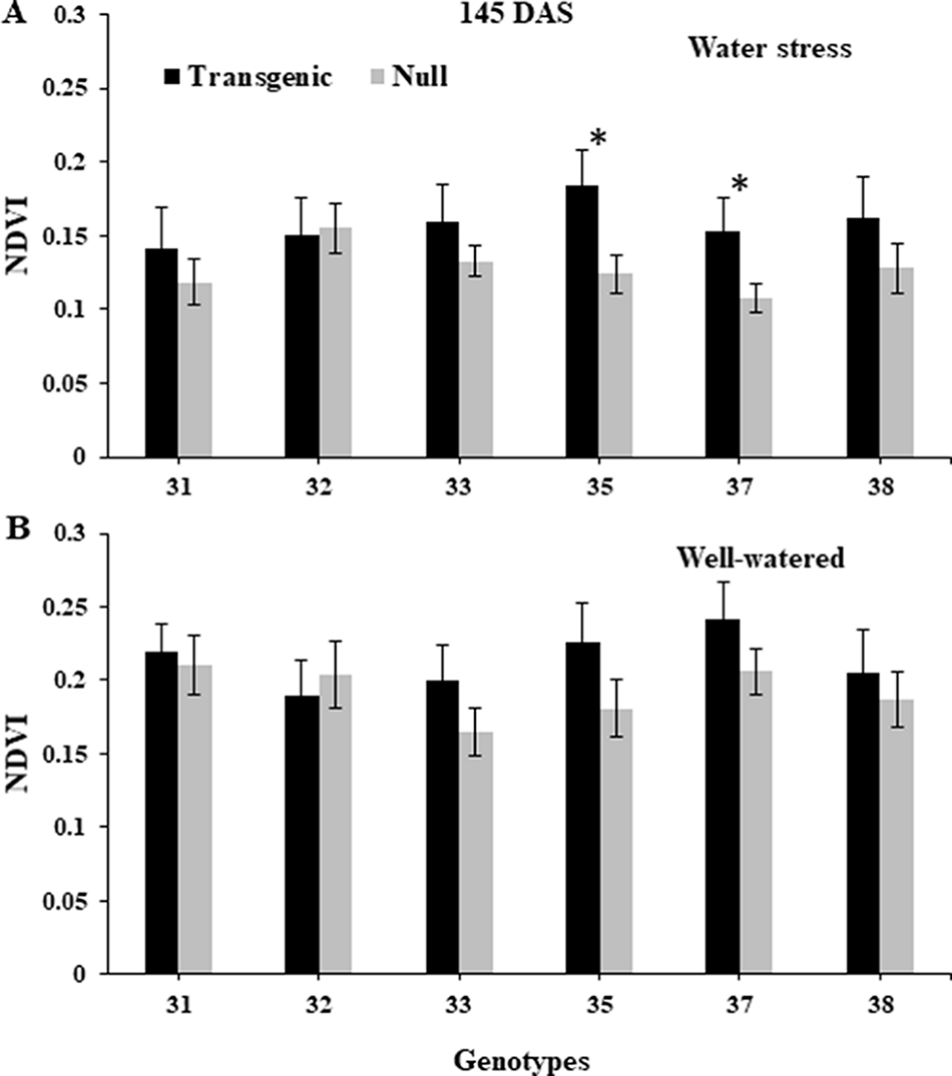
Normalized difference vegetation index (NDVI) of wheat genotypes during post-anthesis under water stressed and well-watered conditions. Data taken at 145 days after sowing (DAS) during the 2014 for water stress **(A)** and well-watered **(B)** treatments. NDVI data was collected using Crop Circle. Data represented as mean ± SEM. *Significant differences at P ≤ 0.05.

**Figure 4:**
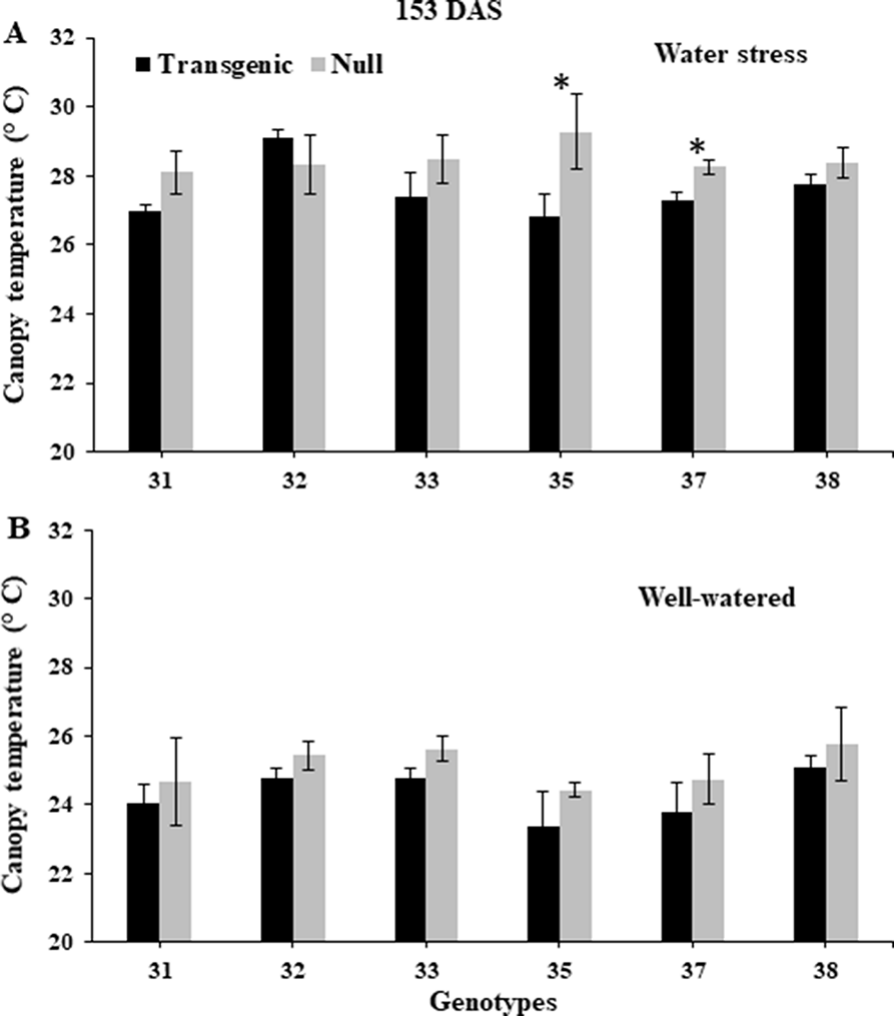
Canopy temperature of wheat genotypes during early grain-filling under water stressed and well-watered conditions. Data taken at 153 days after sowing (DAS) during the 2014 for water stress **(A)** and well-watered **(B)** treatments. Data was collected using a hand-held infra-red thermometer. Data represented as mean ± SEM. *Significant differences at P ≤ 0.05.

NDVI, as an estimate of green biomass, was measured at 13 time points from tillering (Z25) to physiological maturity (Z90). In both 2014 and 2015, well-watered plants mostly showed higher NDVI values compared to water stressed plants, especially during and post-anthesis stages i.e. Z60 onwards (**Figure 2**). Post-anthesis (Z65), transgenic events 35 and 37 showed significantly higher NDVI values compared to corresponding nulls under water stress conditions but were not significantly different under well-watered conditions (**Figure 3** and **Supplementary Figure 6**). Generally, plants in the well-watered treatment demonstrated lower canopy temperatures compared to those in the water stress treatment. It was consistently shown in both experiments that transgenic events 35 and 37 exhibited significantly lower canopy temperatures compared to their corresponding nulls under water stress treatment. Canopy temperatures were not found to be different between transgenic events and nulls under the well-watered treatment (**Figure 4** and **Supplementary Figure 7**).

### Transgenic Plants Had Higher Leaf Water Potential

**Figure 5:**
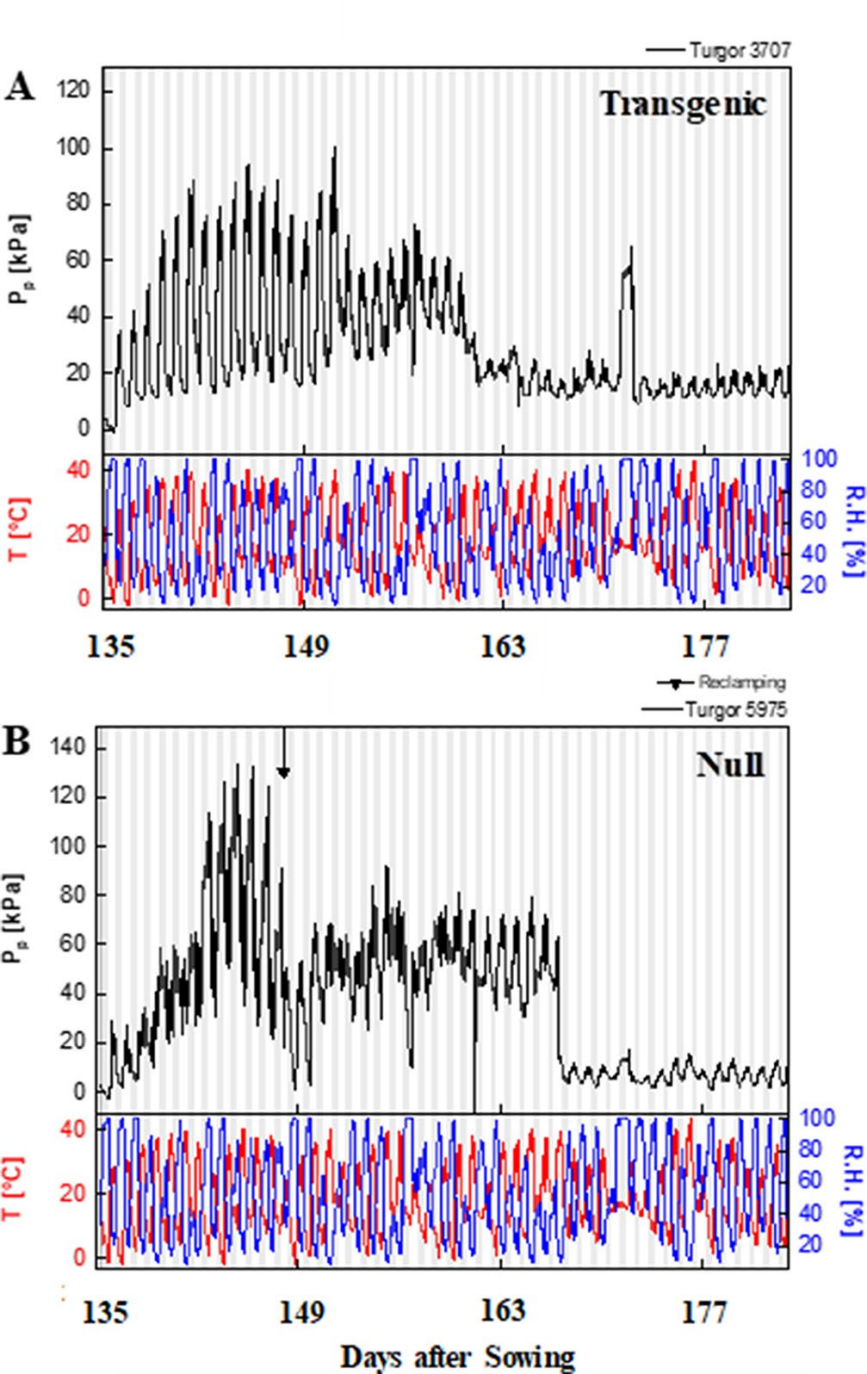
Patch pressure (P_P_) of representative event 37 plants during reproductive phase. Patch pressure (kPa) and weather data collected for a representative transgenic **(A)** and null **(B)** plants during the 2015 field experiment under water stress treatment. P_p_ was measured using Leaf Patch Clamp Pressure (LPCP) probes clamped on flag leaves. White and gray vertical bars represent day and night, respectively. T, temperature (°C); RH, relative humidity (%).

The typical curve behavior of P_p_ measured by LPCP probes should peak during the day and trough at night. Given that P_p_ is inversely proportional to turgor pressure, P_p_ peaks at mid-day, when water loss cannot be compensated by an appropriate uptake of water by the roots. An increase in P_p_ was observed as the season progressed due to an increased loss of leaf turgor pressure, caused by the reduced plant available water as the soil moisture reduced. Temperature and relative humidity measurements were also obtained from the experimental plots, with diurnal changes consistent throughout the experimental period i.e. high temperature and low humidity during the day, and *vice versa* at night (**Figure 5** and **Supplementary Figure 8**). Increases in P_p_ correlated with increases in ambient temperature during the time of day and as temperature increased during the season. During reproductive growth, there was a clear difference observed in the peak P_p_ values with transgenic events 35 (**Supplementary Figure 8**) and 37 (**Figure 5**) compared to their null. P_p_ values reached a peak of 130 kPa in the null plants compared to 100 kPa in the transgenic event 37 plants. Transgenic plants were also able to maintain P_p_ levels for a longer clear peak and trough pattern, with lower nightly P_p_ levels (**Figure 5**). Low P_p_ values were recorded in the transgenic event 35 wherein the P_p_ values reached a peak of 60 kPa while in null plants a peak P_p_ value of 90 kPa was observed (**Supplementary Figure 8**). These trends indicated that transgenic plants were able to maintain higher leaf water status and recover turgor pressure (lower P_p_) at night for longer, under water stress conditions. The ability of transgenic plants to maintain higher leaf water status for longer was also shown by the differences in the time to peak and the relaxation (time to reach the trough). Water stressed transgenic events 33 and 35 reached their peak P_p_ values significantly earlier, in part due to the higher leaf water status (lower P_p_) in these plants at the start of the day (**Table 2**). The difference in relaxation times between transgenic and null plants, such as with transgenic event 37, showed a significantly faster recovery from turgor pressure losses under both well-watered and water stress conditions. This suggested two different mechanisms or responses resulting in the same outcome, transgenic plants maintaining higher leaf water potential.

**Table 2:** Leaf patch pressure (P_p_) in wheat genotypes during reproductive phase in 2015 field experiment.

Treatment	Genotype	Time to peak (min)	Relaxation time (min)
Well-watered	33T	519 ± 27	269 ± 20
	33N	513 ± 30	222 ± 28
	35T	418 ± 20	217 ± 24
	35N	486 ± 18	200 ± 20
	37T	512 ± 36	167 ± 25**
	37N	361 ± 21	267 ± 26
Water stress	33T	511 ± 26*	210 ± 18
	33N	433 ± 37	187 ± 22
	35T	645 ± 30***	129 ± 12
	35N	501 ± 22	145 ± 20
	37T	504 ± 26	167 ± 21***
	37N	556 ± 24	321 ± 24

Time to peak is the number of minutes for the peak reading to be reached from sunrise, and the relaxation time is the number of minutes after sunset to reach 66% of lowest value. Data represented as mean ± SEM. T, transgenic; N, null; *significant differences at P < 0.05; **significant differences at P < 0.01; ***significant differences at P < 0.001.

### Transgenic Plants Showed Improved Grain Yield and Sustained Grain Quality

**Figure 6:**
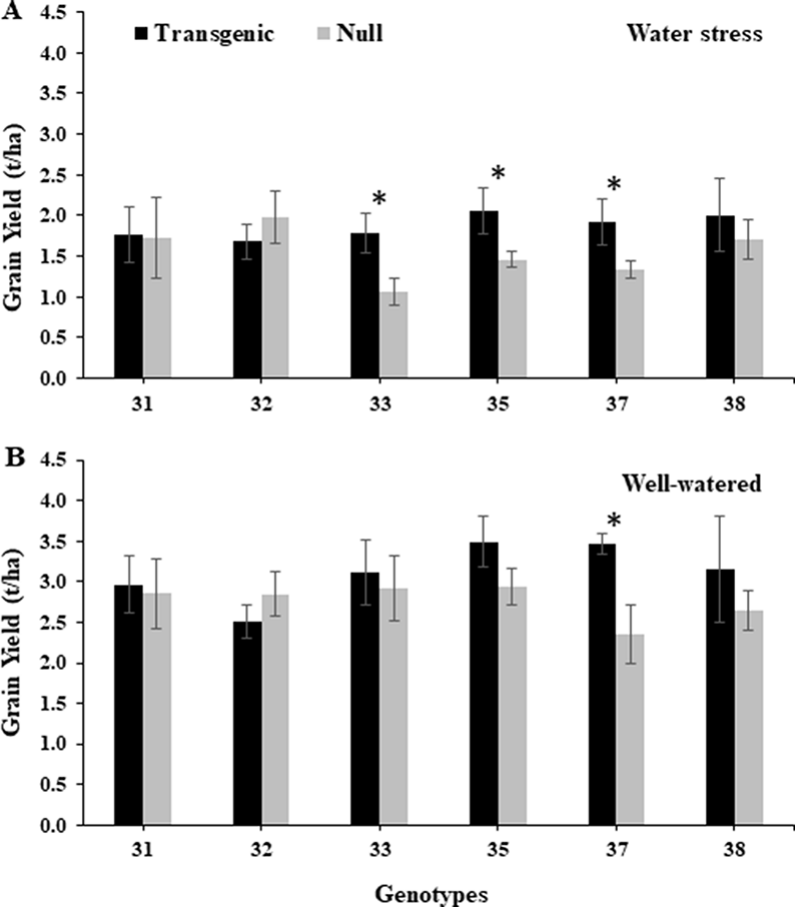
Grain yield of wheat genotypes under water stressed and well-watered conditions. Yield (t/ha) from water stressed **(A)** and well-watered **(B)** treatments from the 2014 field experiment. Data represented as mean ± SEM. *Significant differences at P ≤ 0.05.

Under the water stress treatment during 2014, transgenic events 33, 35, and 37 had a significantly higher grain yield, 40–67%, compared to their corresponding nulls (**Figure 6**). The same transgenic events also showed higher grain yield under the well-watered treatment, although only event 37 in both years and 35 in 2015 showed statistically significant higher yields (**Figure 6** and **Supplementary Figure 9**). Similarly, in 2015, transgenic events 35 and 37 had significantly higher grain yield compared to their corresponding null plants under the water stress treatment (**Supplementary Figure 9**). The grain quality traits, such as grain hardness, test weight, dough stability, and dough extensibility from transgenic grain, were similar to nulls under both water treatments and experiment years. However, lower protein content was observed in transgenic events 33, 35, and 37 (**Supplementary Tables 3A, B and 4A, B**).

**Figure 7:**
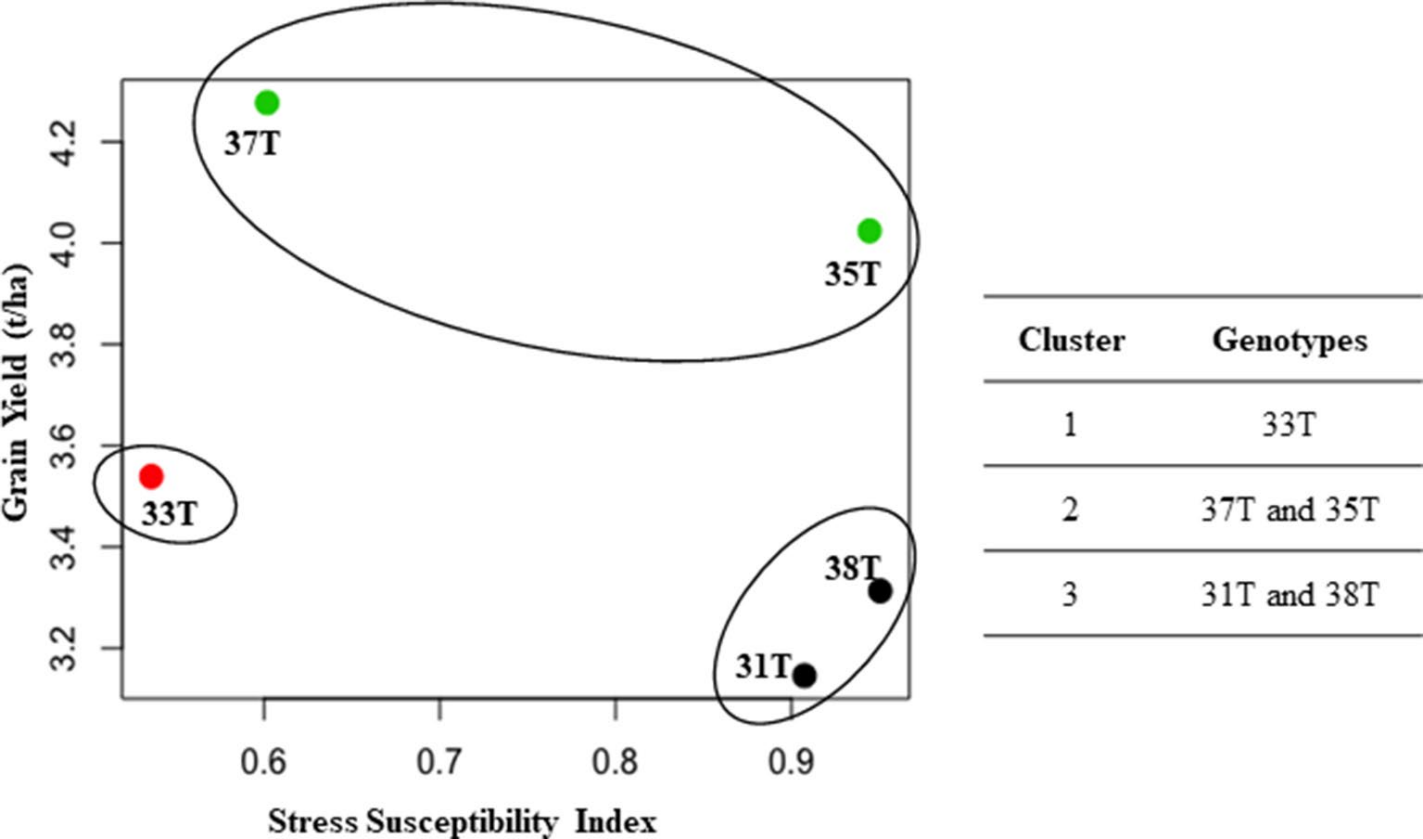
Cluster analysis between grain yield and stress susceptibility index (SSI). Three clusters formed to show relationship between SSI and grain yield (t/ha) (under well-watered conditions). Data represents average of both 2014 and 2015. T, transgenic.

A high positive correlation (r = 0.78) was observed between grain yield and canopy green cover (data not shown). A negative correlation was observed between grain yield and stress susceptibility index (r = −0.8) and between grain yield and canopy temperature (r = −0.45) (data not shown). Cluster analysis was performed using the grain yield from plants grown under well-watered conditions and SSI to elucidate the drought tolerance level of each genotype. Those with a lower SSI were classed as more drought tolerant. The analysis showed three clusters: cluster I, medium yield with high drought tolerance (33T); cluster II, high yield with high drought tolerance (37T and 35T); and cluster III, low yield with low drought tolerance (31T and 38T) (**Figure 7**). Even though event 35T showed poor SSI, a high grain yield was obtained under both treatments; therefore, the event outperformed other events which had a lower SSI, putting it in cluster II.

## Discussion

Climate change predictions have forecasted an increase in the global temperatures and drought events leading to shortages of water for the cultivation of crops (IPCC, 2014). Water stress significantly reduces growth and accelerates leaf senescence due to the reduced time to translocate metabolites from leaves to grain, which ultimately affect grain yield and quality. Delayed leaf senescence can therefore facilitate plants in remobilizing nutrients from old senescing leaves to young leaves and the developing grains (Gregersen et al., 2008). To breed crops that can sustain yield under water limited conditions, it is essential to simulate water stress conditions in field environments. However, inconsistent patterns of rainfall can make it difficult to induce precise water stress treatments, leading to the utilization of novel, custom rainout shelters (Kant et al., 2017). The object of this study was to evaluate transgenic plants, where *IPT* gene expression was driven by a modified *AtMYB32xs* promoter, developed to produce greater grain yield under both limited and optimum water supplies, using mechanisms associated with delayed leaf senescence. Previously, a delay in leaf senescence was achieved through stay-green trait in peas (Armstead et al., 2007), tomato ((Barry et al., 2008), rice (Jiang et al., 2007), and wheat (Christopher et al., 2016). The stay-green trait has been shown to be beneficial in wheat adaptation under water-limited environment (Christopher et al., 2016; Christopher et al., 2018). The introgression of this trait into elite genetic background through conventional breeding might introduce a significant linkage drag in the form of alien alleles and deleterious genes (Schouten et al., 2006; Jacobsen and Schouten, 2009). Also, several back crosses would be needed to reduce this linkage drag (Hasan et al., 2015). To avoid the transfer of unwanted DNA, it may be worth to explore tools which transfers only gene of interest and transgene technology is one such approach.

In our study, the *AtMYB32xs* promoter probably produced a different *IPT* expression profile in transgenic wheat plants compared to previous studies, allowing for optimal function under the well-watered conditions as well. Transgenic tobacco, cotton, peanuts, rice, and wheat plants have been previously transformed using the *IPT* gene with several inducible promoters such as *HSP70*, *SEE1*, *SAG12*, or *SARK* (Medford et al., 1989; Gan and Amasino, 1995; Jordi et al., 2000; Cowan et al., 2005; Rivero et al., 2007; Sýkorová et al., 2008; Peleg et al., 2011; Qin et al., 2011). Due to the stress or inducible nature of such promoters, these studies showed delayed leaf senescence and drought tolerance, but varied with only some resulting in a yield increase only under water and nutrient limited conditions. Indeed, the promoters *SAG12* and *SEE1* promoters have been reported to induce delayed leaf senescence, but with no yield benefits (Robson et al., 2004; Sýkorová et al., 2008). This lack of yield benefit was likely attributed to the failure of metabolites to be translocated from senescing leaves to grains, due to the mechanisms of enhanced delayed leaf senescence that also delayed nutrient remobilization (Sýkorová et al., 2008). Therefore, the selection of the optimal promoter to regulate *IPT* gene expression is critical for obtaining all the benefits, without any deleterious effects. This work follows on from our first research publication in canola (Kant et al., 2015), which has shown that also in wheat the *AtMYB32xs-p::IPT* transgene can induce delayed leaf senescence and enhanced grain yield under both available water conditions.

No abnormalities in morphology and phenology were observed in transgenic plants. The transgenic lines 33, 35, and 37 displayed delayed leaf senescence; however, time to heading and maturity was similar to null lines. This indicates that the transgenic events had no phenological penalties. Delayed leaf senescence in transgenic events 33, 35, and 37 corroborated with higher canopy green coverage (as higher NDVI values) in these transgenic events during post-anthesis. These three transgenic events also showed delayed leaf senescence in the detached leaf assay and had higher grain yield in field experiments (except event 33), which further indicate that the delayed leaf senescence and higher canopy green coverage were consistent to grain yield. Sensor based techniques, such as NDVI, have been previously used to correlate and predict yield with high accuracy in rice (Liu et al., 2017) and wheat (Nguyen et al., 2016). Canopy temperature has been previously used as an indicator to assess crop water stress and to schedule irrigation events (Clawson and Blad, 1982; Dejonge et al., 2015). Indeed, a high correlation between canopy temperature and leaf water potential (r = 0.946) at vegetative, reproductive, and maturation stages in maize suggested that canopy temperature strongly correlated with plant performance (Dejonge et al., 2015). Plants that were well-watered displayed lower canopy temperature and therefore maintained more optimal growth conditions. Lower canopy temperatures in transgenic plants suggested an improved tolerance to water stress, which was found consistent with their higher grain yield. Furthermore, cluster analysis of the SSI in relation to well-watered grain yield indicated that an improvement in the level of drought tolerance for transgenic events was achieved, especially for events 35 and 37.

The maintenance of higher leaf water potential is important for improved plant growth and efficient source–sink relationships which can translate to higher crop yield. LPCP probes were used to measure P_P_, which is inversely proportional to leaf water potential (Kant et al., 2014), and was lower in transgenic events 33, 35, and 37. Specifically, transgenic events 33 and 35 were shown to reach peak P_P_ values later in the day, while transgenic event 37 had a shorter relaxation time, which indicated a faster recovery from turgor pressure loss, and the longer maintenance of water potential. The delay in having reached peak P_P_ and the shorter recovery time observed was consistent with improved leaf water status, which corresponded with higher yields. In this study, the improved leaf water potential, chlorophyll levels, and biomass corresponded with improved grain yield, which was consistent with transgenic canola reported by Kant et al. (2015). The increase in grain yield found in transgenic wheat plants was 26% to 67%, under water stress conditions, compared to well-watered transgenic plants, where the grain yield increase was less pronounced, of only 6 to 16%. This showed that the *AtMYB32xs-p::IPT* transgene was effective during reproductive and grain filling stage. The yield increase was probably the result of re-programmed cytokinin biosynthesis that facilitated remobilization of nutrients and improved the efficiency of the source–sink relationship. This study was consistent with the higher grain yield shown with water-stressed transgenic rice that was engineered with the *SARK-p::IPT* transgene (Reguera et al., 2013).

The differential expression of *IPT* gene was observed among transformants. However, transgenic events 32 and 38 displayed notable *IPT* gene expression, and failed to show any growth and yield advantage. Event 35 had highest gene expression coupled with higher grain yield, crop canopy green cover, crop canopy temperature, and leaf water potential. On the other hand, although low expression of *IPT* gene was observed in event 37, the plants showed high grain yield under water stress and well-watered treatments and exhibited good performance in growth related traits. This could be due to the position on the genome where the transgene has been inserted may also influence the timing and tissue specificity of expression other than the expression level, due to the influence of its surrounding genes (Chen and Zhang, 2016; Joshi et al., 2011). Grain quality parameters such as grain hardness, test weight, dough stability, and dough extensibility were similar in grain harvested from transgenic and null plants. However, the grain protein content was lower for three transgenic events exposed to the water stress treatment. The negative correlation between protein and carbohydrate in cereals has been well established, whereby a penalty in protein content has been observed with an increase in grain yield (Terman et al., 1969; Walker et al., 2017).

## Conclusion

In conclusion, transgenic wheat events generated with *AtMYB32xs-p::IPT* transgene showed delayed leaf senescence and retention of chlorophyll for a longer duration. The improved performance of transgenic wheat plants, particularly under water deficit stress, was corroborated through higher canopy green cover, lower canopy temperature, maintenance of leaf water potential, and higher grain yield. This transgenic technology has potential high value to wheat and other crop species to improve water use efficiency and reduce crop loss induced by water deficit with no yield penalty under optimal water availability and growth conditions. The use of the *AtMYB32xs-p::IPT* transgene to improve grain yield in both canola and wheat showed that a developmentally regulated increase in cytokinin is a potential novel approach for crop breeding.

## Data Availability Statement

All datasets generated for this study are included in the manuscript/**Supplementary Files**.

## Author Contributions

GS: conceived the idea. SK, SJ: designed the field experiments. SJ, AC: conducted the field experiments. SJ, AC, SK, DI, JP: conducted laboratory experiments and analyzed the data. SJ, AC, SK, DI, JP, GS: interpreted results and wrote the paper. All authors read and approved the final manuscript.

## Conflict of Interest

The authors declare that the research was conducted in the absence of any commercial or financial relationships that could be construed as a potential conflict of interest.

## Supplementary Material

The Supplementary Material for this article can be found at: https://www.frontiersin.org/articles/10.3389/fpls.2019.01285/full#supplementary-material
