# Supplementary material for: Improved Wheat Growth and Yield by Delayed Leaf Senescence Using Developmentally Regulated Expression of a Cytokinin Biosynthesis Gene
Source: Front Plant Sci. 2019 Oct 18;10:1285. doi: 10.3389/fpls.2019.01285 (PMC6813231; doi:10.3389/fpls.2019.01285)
Supplement: Supplementary file 1 [file Table_1.docx]

**Supplementary Table 1.** Details of primers used in RT-PCR.

| Primers | Sequence (5ʹ > 3 ʹ) | Product length (bp) |
| --- | --- | --- |
| *Actin*-Forward | AGCTCGCATATGTGGCTCTT | 340 |
| *Actin*-Reverse | TTGATCTTCATGCTGCTTGG |  |
| *IPT*-1-Forward | GGTGGAGGGTATCATCGCAG | 121 |
| *IPT*-1-Reverse | CATGCAGTTGAGCAACGAGG |  |

**Supplementary Table 2.** Rainfall received during 2014 and 2015 field experiments, along with the long-term

averages for Horsham, Victoria, Australia. 2014, 2015 and long-term weather data from weather station 79100 (<http://www.bom.gov.au/climate>).

|  | May | June | July | August | September | October | November | December | Total |
| --- | --- | --- | --- | --- | --- | --- | --- | --- | --- |
| 2014 | 32.5 | 44.6 | 35.8 | 9.8 | 15.2 | 4.8 | 16.0 | 11.8 | 170.5 |
| 2015 | 20.0 | 34.6 | 18.0 | 8.6 | 26.6 | 2.6 | 17.0 | 12.0 | 139.4 |
| Long-term average | 32.2 | 37.9 | 41.2 | 39.3 | 37.6 | 31.7 | 29.7 | 25.6 | 275.2 |

**Supplementary Table 3A**. Grain quality parameters: protein, grain hardness and test weight in wheat genotypes from 2014 field experiment.

| Genotypes | Protein (%) | | |  | Grain hardness (%) | | |  | Test weight (g) | | |
| --- | --- | --- | --- | --- | --- | --- | --- | --- | --- | --- | --- |
|  | **Well-watered** | **Water stress** | Average |  | **Well-watered** | **Water stress** | Average |  | **Well-watered** | **Water stress** | Average |
| 31T | 11.1 | 11.6 | 11.3 |  | 15.9 | 16.8 | 16.4 |  | 83.5 | 81.9 | 82.7 |
| 31N | 10.9 | 12.4 | 11.7 |  | 17.1 | 15.9 | 16.5 |  | 82.8 | 82.1 | 82.5 |
| 32T | 11.2 | 11.5 | 11.4 |  | 18.6 | 14.4 | 16.5 |  | 82.9 | 83.8 | 83.4 |
| 32N | 10.4 | 11.7 | 11.0 |  | 18.0 | 15.3 | 16.7 |  | 83.9 | 82.8 | 83.4 |
| 33T | 10.4 | 10.2 | 10.3 |  | 18.3 | 15.9 | 17.1 |  | 84.0 | 83.6 | 83.8 |
| 33N | 10.8 | 11.7 | 11.2 |  | 17.6 | 14.9 | 16.3 |  | 83.2 | 82.3 | 82.8 |
| 35T | 11.7 | 11.2 | 11.4 |  | 18.6 | 14.8 | 16.7 |  | 82.2 | 83.7 | 83.0 |
| 35N | 11.4 | 12.1 | 11.7 |  | 19.2 | 16.1 | 17.7 |  | 82.7 | 82.3 | 82.5 |
| 37T | 11.1 | 11.4 | 11.2 |  | 14.5 | 15.4 | 15.0 |  | 85.3 | 83.5 | 84.4 |
| 37N | 11.4 | 12.3 | 11.9 |  | 16.0 | 15.4 | 15.7 |  | 84.3 | 81.5 | 82.9 |
| 38T | 10.5 | 11.1 | 10.8 |  | 18.0 | 14.6 | 16.3 |  | 83.0 | 83.5 | 83.3 |
| 38N | 10.9 | 9.9 | 10.4 |  | 19.0 | 15.9 | 17.5 |  | 82.9 | 84.3 | 83.6 |
| Bobwhite | 11.3 | 11.6 | 11.2 |  | 17.3 | 15.3 | 16.3 |  | 83.9 | 84.2 | 84.1 |
| Statistics | Irrigation | Genotype | Irrigation*  Genotype |  | Irrigation | Genotype | Irrigation*  Genotype |  | Irrigation | Genotype | Irrigation*Genotype |
| SED | 0.2263 | 0.3841 | 0.5696 |  | 0.675 | 0.967 | 1.478 |  | 0.565 | 0.691 | 1.097 |
| LSD (5%) | 0.7201 | 0.7701 | 1.1464 |  | 2.149 | 1.938 | 2.992 |  | 1.799 | 1.384 | 2.240 |
| P value | 0.390 | 0.005 | 0.384 |  | 0.069 | 0.499 | 0.232 |  | 0.455 | 0.358 | 0.265 |

SED, standard error of difference; LSD, least significant difference; T, transgenic; N, null.

**Supplementary Table 3B**. Grain quality parameters: dough stability and dough extensibility

in wheat genotypes from 2014 field experiment.

| Genotypes | Dough Stability (BU) | | |  | Dough Extensibility (cm) | | |
| --- | --- | --- | --- | --- | --- | --- | --- |
|  | **Well-watered** | **Water stress** | Average |  | **Well-watered** | **Water stress** | Average |
| 31T | 6.7 | 6.9 | 6.8 |  | 22.3 | 22.0 | 22.2 |
| 31N | 6.2 | 8.5 | 7.4 |  | 22.5 | 22.3 | 22.4 |
| 32T | 6.1 | 6.9 | 6.5 |  | 21.8 | 21.8 | 21.8 |
| 32N | 6.0 | 7.4 | 6.7 |  | 21.5 | 21.8 | 21.7 |
| 33T | 5.9 | 5.5 | 5.7 |  | 21.4 | 21.2 | 21.3 |
| 33N | 6.5 | 6.9 | 6.7 |  | 21.4 | 21.6 | 21.5 |
| 35T | 6.6 | 6.9 | 6.8 |  | 22.5 | 22.1 | 22.3 |
| 35N | 6.3 | 8.3 | 7.3 |  | 22.3 | 22.2 | 22.3 |
| 37T | 6.2 | 7.5 | 6.9 |  | 22.0 | 21.8 | 21.9 |
| 37N | 5.8 | 7.2 | 6.5 |  | 22.5 | 21.9 | 22.2 |
| 38T | 6.0 | 7.3 | 6.7 |  | 21.7 | 22.0 | 21.9 |
| 38N | 6.2 | 6.3 | 6.3 |  | 22.0 | 21.5 | 21.8 |
| Bobwhite | 6.5 | 6.8 | 6.7 |  | 22.1 | 23.0 | 22.6 |
| Statistics | Irrigation | Genotype | Irrigation*Genotype |  | Irrigation | Genotype | Irrigation*Genotype |
| SED | 0.2269 | 0.4416 | 0.6423 |  | 0.2019 | 0.4054 | 0.5875 |
| LSD (5%) | 0.7221 | 0.8853 | 1.2891 |  | 0.6426 | 0.8128 | 1.1787 |
| P value | 0.307 | 0.307 | 0.817 |  | 0.138 | 0.227 | 0.751 |

SED, standard error of difference; LSD, least significant difference; T, transgenic; N, null.

**Supplementary Table 4A**. Grain quality parameters: protein, grain hardness and test weight in wheat genotypes from 2015 field experiment.

| Genotypes | Protein (%) | | |  | Grain hardness (%) | | |  | Test weight (g) | | |
| --- | --- | --- | --- | --- | --- | --- | --- | --- | --- | --- | --- |
|  | **Well-watered** | **Water stress** | Average |  | **Well-watered** | **Water stress** | Average |  | **Well-watered** | **Water stress** | Average |
| 31T | 12.7 | 12.1 | 12.4 |  | 16.1 | 15.8 | 16.0 |  | 79.8 | 78.5 | 79.2 |
| 31N | 12.8 | 12.3 | 12.6 |  | 17.0 | 16.0 | 16.5 |  | 80.2 | 80.1 | 80.2 |
| 33T | 12.3 | 11.7 | 12.0 |  | 15.9 | 16.4 | 16.2 |  | 79.2 | 80.8 | 80.0 |
| 33N | 12.5 | 12.0 | 12.3 |  | 16.5 | 16.2 | 16.4 |  | 79.8 | 80.4 | 80.1 |
| 35T | 12.7 | 11.1 | 11.9 |  | 16.8 | 16.2 | 16.5 |  | 80.9 | 79.5 | 80.2 |
| 35N | 12.4 | 11.6 | 12.0 |  | 16.7 | 16.8 | 16.8 |  | 80.8 | 81.0 | 80.9 |
| 37T | 13.3 | 11.3 | 12.3 |  | 16.4 | 16.8 | 16.6 |  | 80.6 | 80.6 | 80.6 |
| 37N | 14.0 | 15.1 | 14.6 |  | 16.3 | 16.5 | 16.4 |  | 79.7 | 78.7 | 79.2 |
| 38T | 12.1 | 11.5 | 11.8 |  | 16.4 | 16.5 | 16.5 |  | 79.5 | 80.4 | 80.0 |
| 38N | 12.6 | 12.0 | 12.3 |  | 16.4 | 16.5 | 16.5 |  | 79.2 | 80.3 | 79.8 |
| Bobwhite | 12.3 | 12.3 | 12.3 |  | 16.4 | 16.8 | 16.6 |  | 79.3 | 79.4 | 79.4 |
| Statistics | Irrigation | Genotype | Irrigation*Genotype |  | Irrigation | Genotype | Irrigation*Genotype |  | Irrigation | Genotype | Irrigation*Genotype |
| SED | 0.0548 | 0.2993 | 0.4028 |  | 0.1897 | 0.3134 | 0.4589 |  | 0.230 | 0.436 | 0.625 |
| LSD (5%) | 0.1744 | 0.4070 | 0.8160 |  | 0.6036 | 0.6356 | 0.9319 |  | 0.731 | 0.884 | 1.266 |
| P value | 0.062 | <0.001 | 0.225 |  | 0.429 | 0.405 | 0.332 |  | 0.521 | 0.501 | 0.123 |

SED, standard error of difference; LSD, least significant difference; T, transgenic; N, null.

**Supplementary Table 4B**. Grain quality parameters: dough stability and dough extensibility

in wheat genotypes from 2015 field experiment.

| Genotypes | Dough stability (BU) | | | | Dough extensibility (cm) | | |
| --- | --- | --- | --- | --- | --- | --- | --- |
|  | **Well-watered** | **Water stress** | Average |  | **Well-watered** | **Water stress** | Average |
| 31T | 8.4 | 7.1 | 7.8 |  | 23.7 | 23.4 | 23.6 |
| 31N | 7.6 | 7.8 | 7.7 |  | 23.7 | 23.7 | 23.7 |
| 33T | 7.2 | 7.3 | 7.3 |  | 23.5 | 23.4 | 23.5 |
| 33N | 7.4 | 8.5 | 8.0 |  | 23.6 | 23.5 | 23.6 |
| 35T | 7.5 | 7.2 | 7.4 |  | 23.5 | 23.1 | 23.3 |
| 35N | 7.8 | 7.2 | 7.5 |  | 23.7 | 23.3 | 23.5 |
| 37T | 7.8 | 7.1 | 7.5 |  | 23.4 | 23.2 | 23.3 |
| 37N | 7.9 | 8.2 | 8.1 |  | 23.6 | 24.2 | 23.9 |
| 38T | 8.3 | 7.9 | 8.1 |  | 23.3 | 23.2 | 23.3 |
| 38N | 7.1 | 7.5 | 7.3 |  | 23.7 | 23.2 | 23.5 |
| Bobwhite | 6.3 | 6.0 | 6.2 |  | 23.5 | 23.5 | 23.5 |
| Statistics | Irrigation | Genotype | Irrigation*Genotype |  | Irrigation | Genotype | Irrigation*Genotype |
| SED | 0.2887 | 0.4039 | 0.6110 |  | 0.0449 | 0.1349 | 0.1854 |
| LSD (5%) | 0.9187 | 0.8191 | 1.2479 |  | 0.1430 | 0.2735 | 0.3750 |
| P value | 0.631 | 0.303 | 0.699 |  | 0.064 | 0.401 | 0.296 |

SED, standard error of difference; LSD, least significant difference; T, transgenic; N, null.
