# Supplementary material for: Improved Wheat Growth and Yield by Delayed Leaf Senescence Using Developmentally Regulated Expression of a Cytokinin Biosynthesis Gene
Source: Front Plant Sci. 2019 Oct 18;10:1285. doi: 10.3389/fpls.2019.01285 (PMC6813231; doi:10.3389/fpls.2019.01285)
Supplement: Supplementary file 2 [file Presentation_1.pptx]

## Slide 1
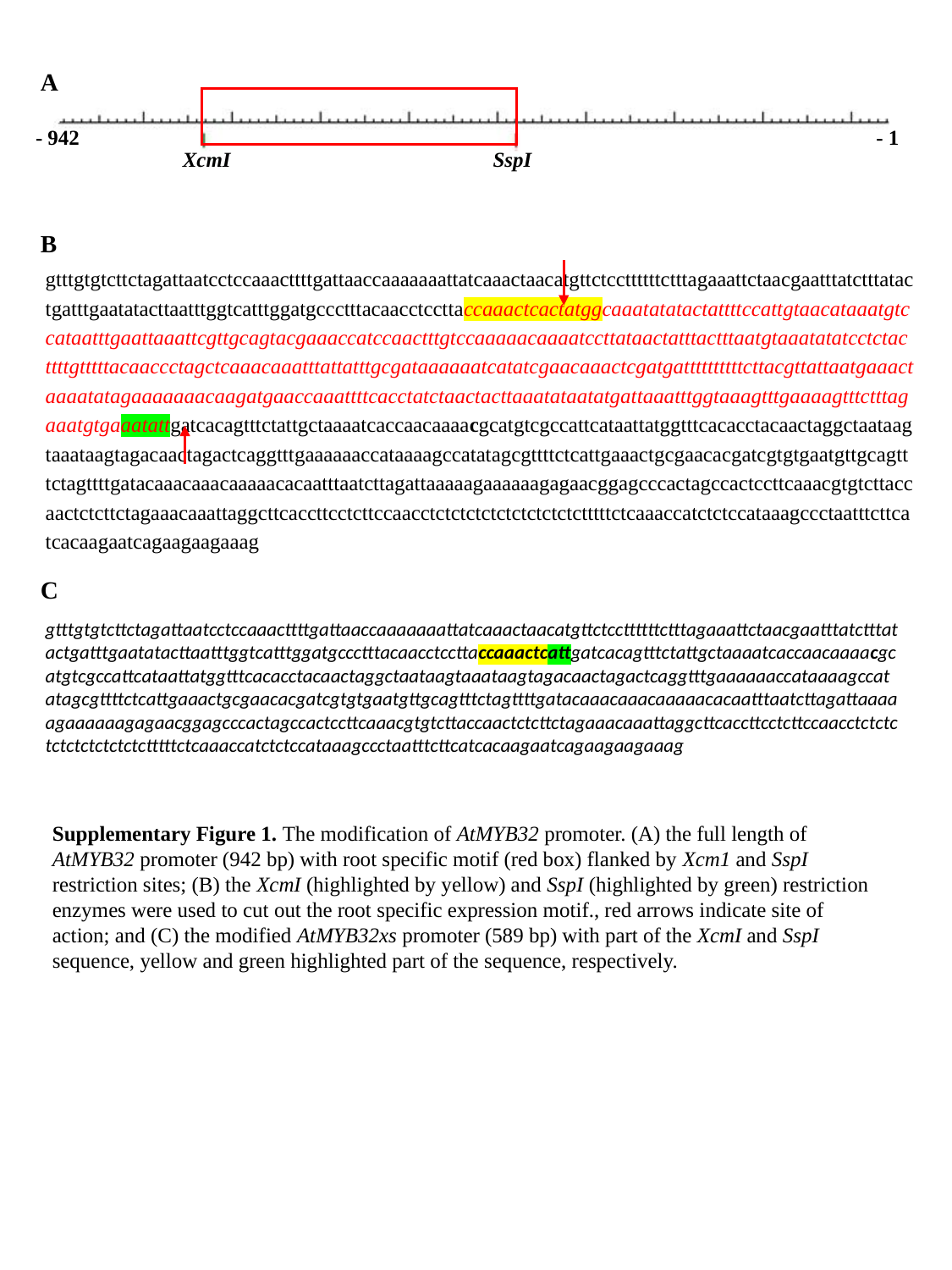

A
- 942
- 1
XcmI
SspI
B
gtttgtgtcttctagattaatcctccaaacttttgattaaccaaaaaaattatcaaactaacatgttctccttttttctttagaaattctaacgaatttatctttatactgatttgaatatacttaatttggtcatttggatgccctttacaacctccttaccaaactcactatggcaaatatatactattttccattgtaacataaatgtccataatttgaattaaattcgttgcagtacgaaaccatccaactttgtccaaaaacaaaatccttataactatttactttaatgtaaatatatcctctacttttgtttttacaaccctagctcaaacaaatttattatttgcgataaaaaatcatatcgaacaaactcgatgattttttttttcttacgttattaatgaaactaaaatatagaaaaaaacaagatgaaccaaattttcacctatctaactacttaaatataatatgattaaatttggtaaagtttgaaaagtttctttagaaatgtgaaatattgatcacagtttctattgctaaaatcaccaacaaaacgcatgtcgccattcataattatggtttcacacctacaactaggctaataagtaaataagtagacaactagactcaggtttgaaaaaaccataaaagccatatagcgttttctcattgaaactgcgaacacgatcgtgtgaatgttgcagtttctagttttgatacaaacaaacaaaaacacaatttaatcttagattaaaaagaaaaaagagaacggagcccactagccactccttcaaacgtgtcttaccaactctcttctagaaacaaattaggcttcaccttcctcttccaacctctctctctctctctctctctttttctcaaaccatctctccataaagccctaatttcttcatcacaagaatcagaagaagaaag
C
gtttgtgtcttctagattaatcctccaaacttttgattaaccaaaaaaattatcaaactaacatgttctccttttttctttagaaattctaacgaatttatctttatactgatttgaatatacttaatttggtcatttggatgccctttacaacctccttaccaaactcattgatcacagtttctattgctaaaatcaccaacaaaacgcatgtcgccattcataattatggtttcacacctacaactaggctaataagtaaataagtagacaactagactcaggtttgaaaaaaccataaaagccatatagcgttttctcattgaaactgcgaacacgatcgtgtgaatgttgcagtttctagttttgatacaaacaaacaaaaacacaatttaatcttagattaaaaagaaaaaagagaacggagcccactagccactccttcaaacgtgtcttaccaactctcttctagaaacaaattaggcttcaccttcctcttccaacctctctctctctctctctctctttttctcaaaccatctctccataaagccctaatttcttcatcacaagaatcagaagaagaaag
Supplementary Figure 1. The modification of AtMYB32 promoter. (A) the full length of AtMYB32 promoter (942 bp) with root specific motif (red box) flanked by Xcm1 and SspI restriction sites; (B) the XcmI (highlighted by yellow) and SspI (highlighted by green) restriction enzymes were used to cut out the root specific expression motif., red arrows indicate site of action; and (C) the modified AtMYB32xs promoter (589 bp) with part of the XcmI and SspI sequence, yellow and green highlighted part of the sequence, respectively.

## Slide 2
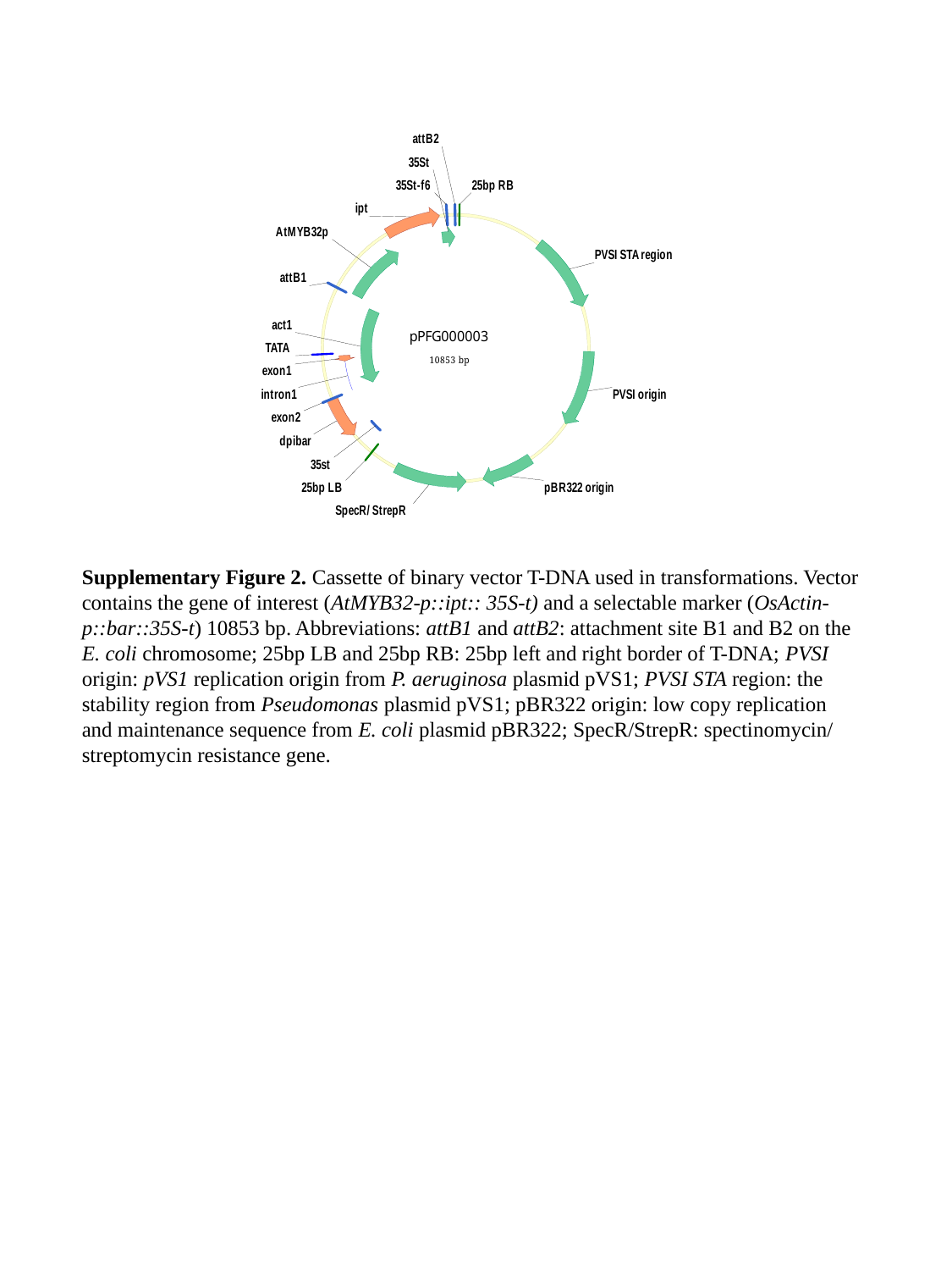

Supplementary Figure 2. Cassette of binary vector T-DNA used in transformations. Vector contains the gene of interest (AtMYB32-p::ipt:: 35S-t) and a selectable marker (OsActin-p::bar::35S-t) 10853 bp. Abbreviations: attB1 and attB2: attachment site B1 and B2 on the E. coli chromosome; 25bp LB and 25bp RB: 25bp left and right border of T-DNA; PVSI origin: pVS1 replication origin from P. aeruginosa plasmid pVS1; PVSI STA region: the stability region from Pseudomonas plasmid pVS1; pBR322 origin: low copy replication and maintenance sequence from E. coli plasmid pBR322; SpecR/StrepR: spectinomycin/ streptomycin resistance gene.

## Slide 3
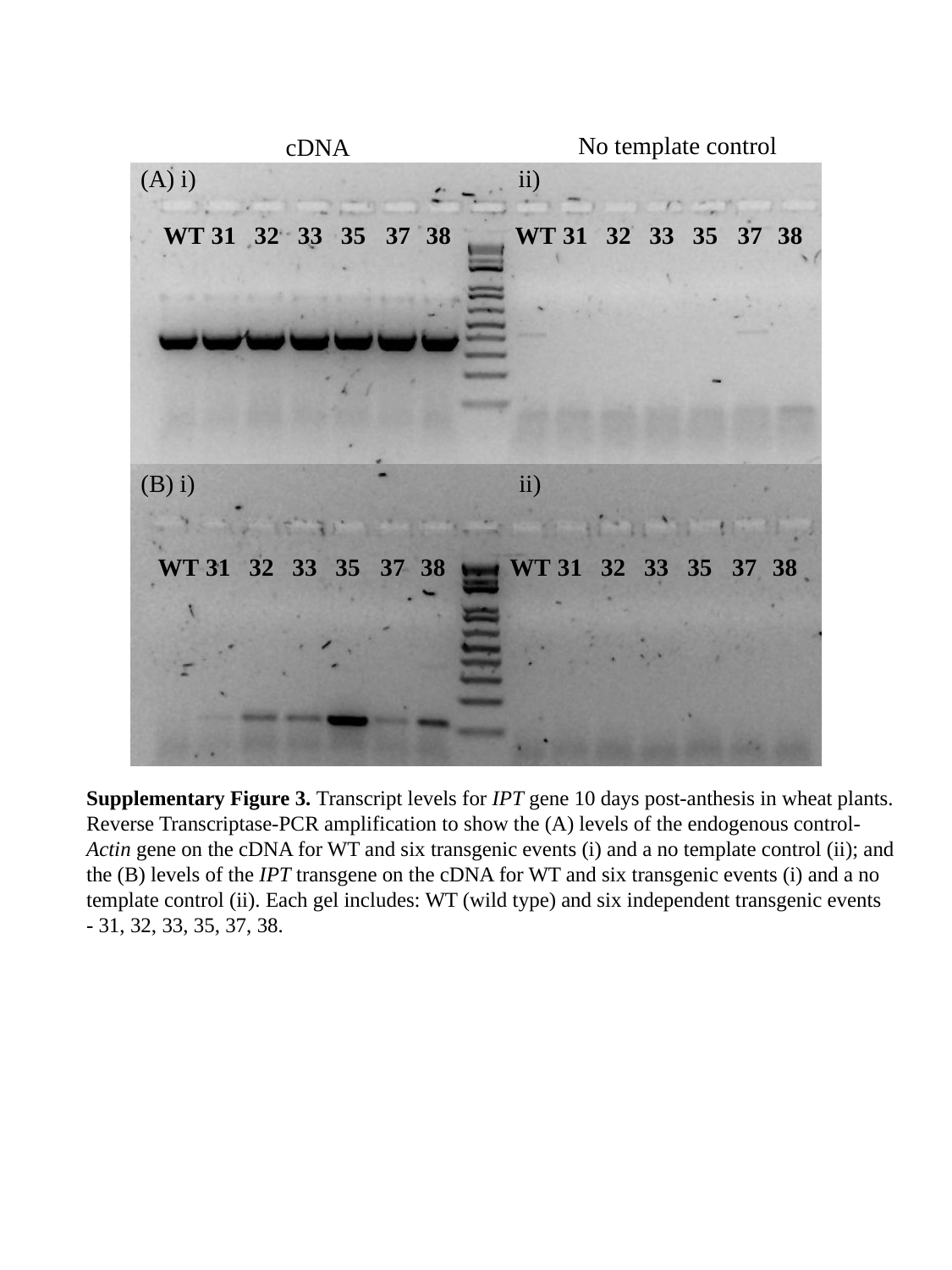

No template control
cDNA
(A) i)
ii)
WT
31
32
33
35
37
38
WT
31
32
33
35
37
38
ii)
(B) i)
WT
31
32
33
35
37
38
WT
31
32
33
35
37
38
Supplementary Figure 3. Transcript levels for IPT gene 10 days post-anthesis in wheat plants. Reverse Transcriptase-PCR amplification to show the (A) levels of the endogenous control-Actin gene on the cDNA for WT and six transgenic events (i) and a no template control (ii); and the (B) levels of the IPT transgene on the cDNA for WT and six transgenic events (i) and a no template control (ii). Each gel includes: WT (wild type) and six independent transgenic events - 31, 32, 33, 35, 37, 38.

## Slide 4
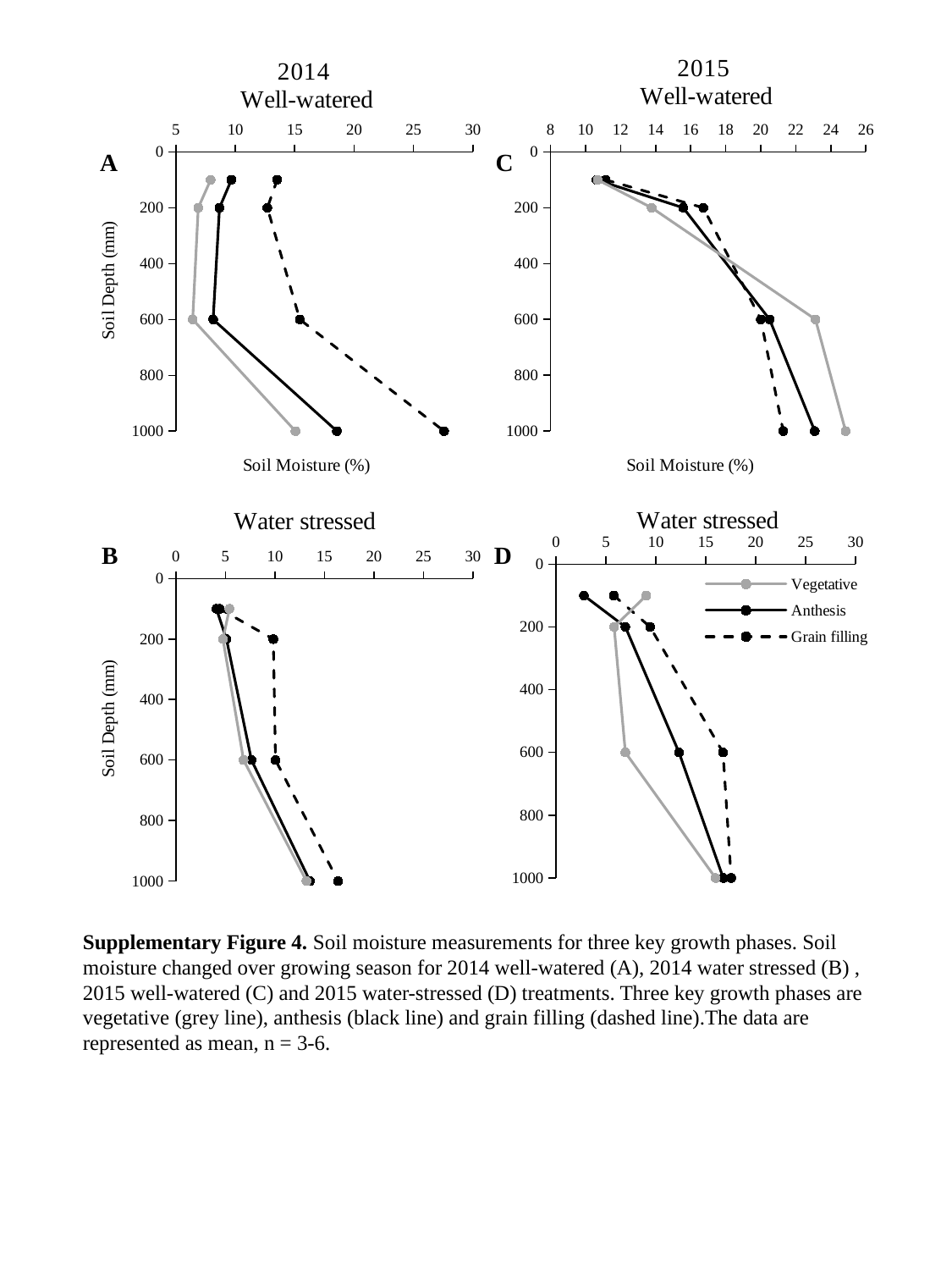

### Chart: 2014
Well-watered
| Category | Vegetative | Flowering | Grain filling |
|---|---|---|---|
### Chart: 2015
Well-watered
| Category | Vegetative | Flowering | Grain filling |
|---|---|---|---|A
C
### Chart: Water stressed
| Category | Vegetative | Flowering | Grain filling |
|---|---|---|---|
### Chart: Water stressed
| Category | Vegetative | | Grain filling |
|---|---|---|---|D
B
Supplementary Figure 4. Soil moisture measurements for three key growth phases. Soil moisture changed over growing season for 2014 well-watered (A), 2014 water stressed (B) , 2015 well-watered (C) and 2015 water-stressed (D) treatments. Three key growth phases are vegetative (grey line), anthesis (black line) and grain filling (dashed line).The data are represented as mean, n = 3-6.

## Slide 5
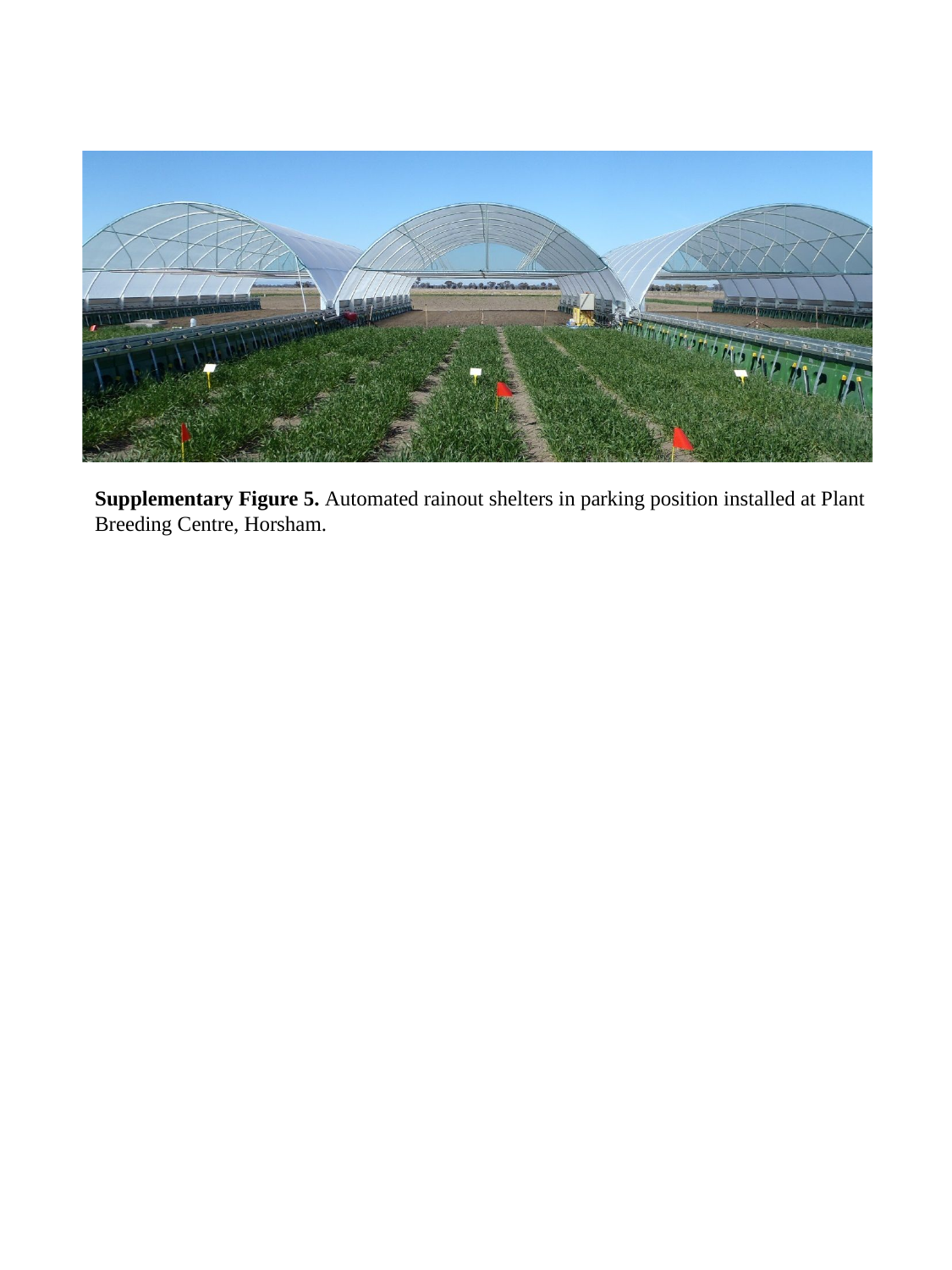

Supplementary Figure 5. Automated rainout shelters in parking position installed at Plant Breeding Centre, Horsham.

## Slide 6
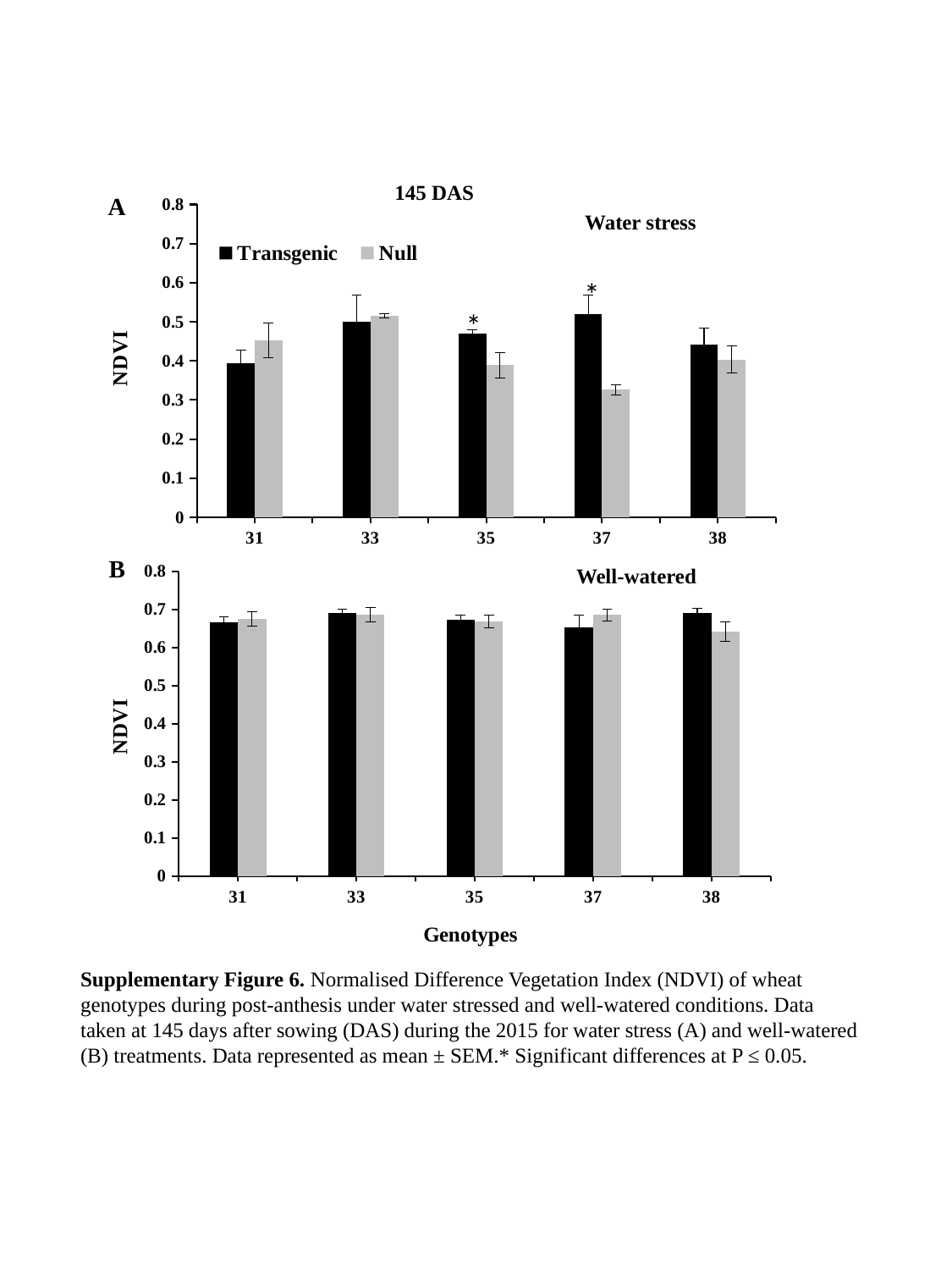

### Chart
| Category | Transgenic | Null |
|---|---|---|
| 31 | 0.39325 | 0.452 |
| 33 | 0.5008 | 0.51525 |
| 35 | 0.4695 | 0.388775 |
| 37 | 0.518866666666667 | 0.32635 |
| 38 | 0.441975 | 0.403125 |145 DAS
A
Water stress
*
*
B
### Chart
| Category | Transgenic | Null |
|---|---|---|
| 31 | 0.66485 | 0.6741 |
| 33 | 0.69115 | 0.68535 |
| 35 | 0.67345 | 0.668025 |
| 37 | 0.652025 | 0.68485 |
| 38 | 0.689125 | 0.64065 |Well-watered
Supplementary Figure 6. Normalised Difference Vegetation Index (NDVI) of wheat genotypes during post-anthesis under water stressed and well-watered conditions. Data taken at 145 days after sowing (DAS) during the 2015 for water stress (A) and well-watered (B) treatments. Data represented as mean ± SEM.* Significant differences at P ≤ 0.05.

## Slide 7
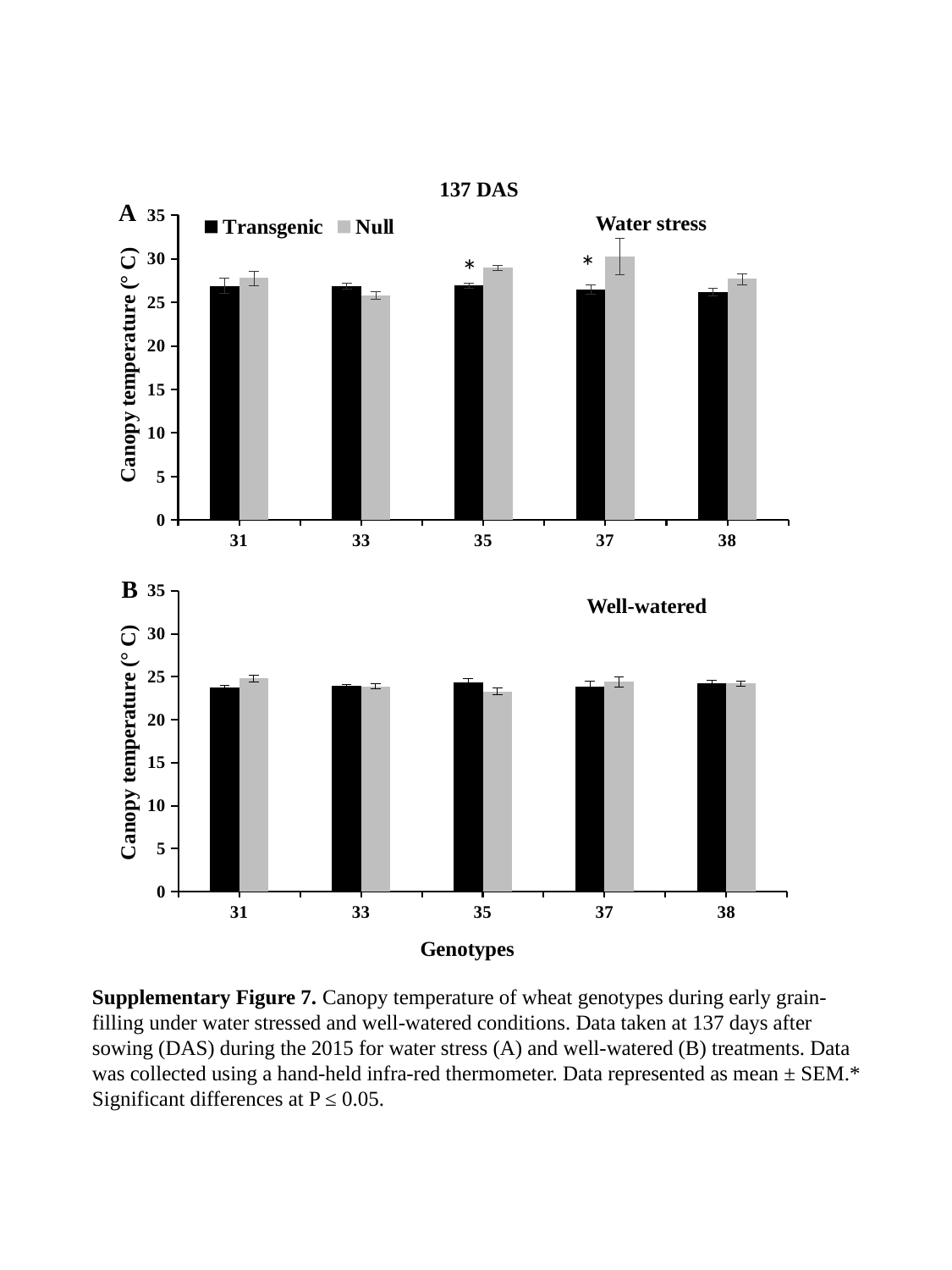

137 DAS
A
### Chart
| Category | Transgenic | Null |
|---|---|---|
| 31 | 26.9 | 27.8 |
| 33 | 26.9 | 25.8 |
| 35 | 26.95 | 28.95 |
| 37 | 26.5 | 30.3 |
| 38 | 26.2 | 27.7 |Water stress
*
*
### Chart
| Category | Transgenic | Null |
|---|---|---|
| 31 | 23.8 | 24.8 |
| 33 | 24.0 | 23.9 |
| 35 | 24.3 | 23.3 |
| 37 | 23.9 | 24.4 |
| 38 | 24.2 | 24.2 |B
Well-watered
Supplementary Figure 7. Canopy temperature of wheat genotypes during early grain- filling under water stressed and well-watered conditions. Data taken at 137 days after sowing (DAS) during the 2015 for water stress (A) and well-watered (B) treatments. Data was collected using a hand-held infra-red thermometer. Data represented as mean ± SEM.* Significant differences at P ≤ 0.05.

## Slide 8
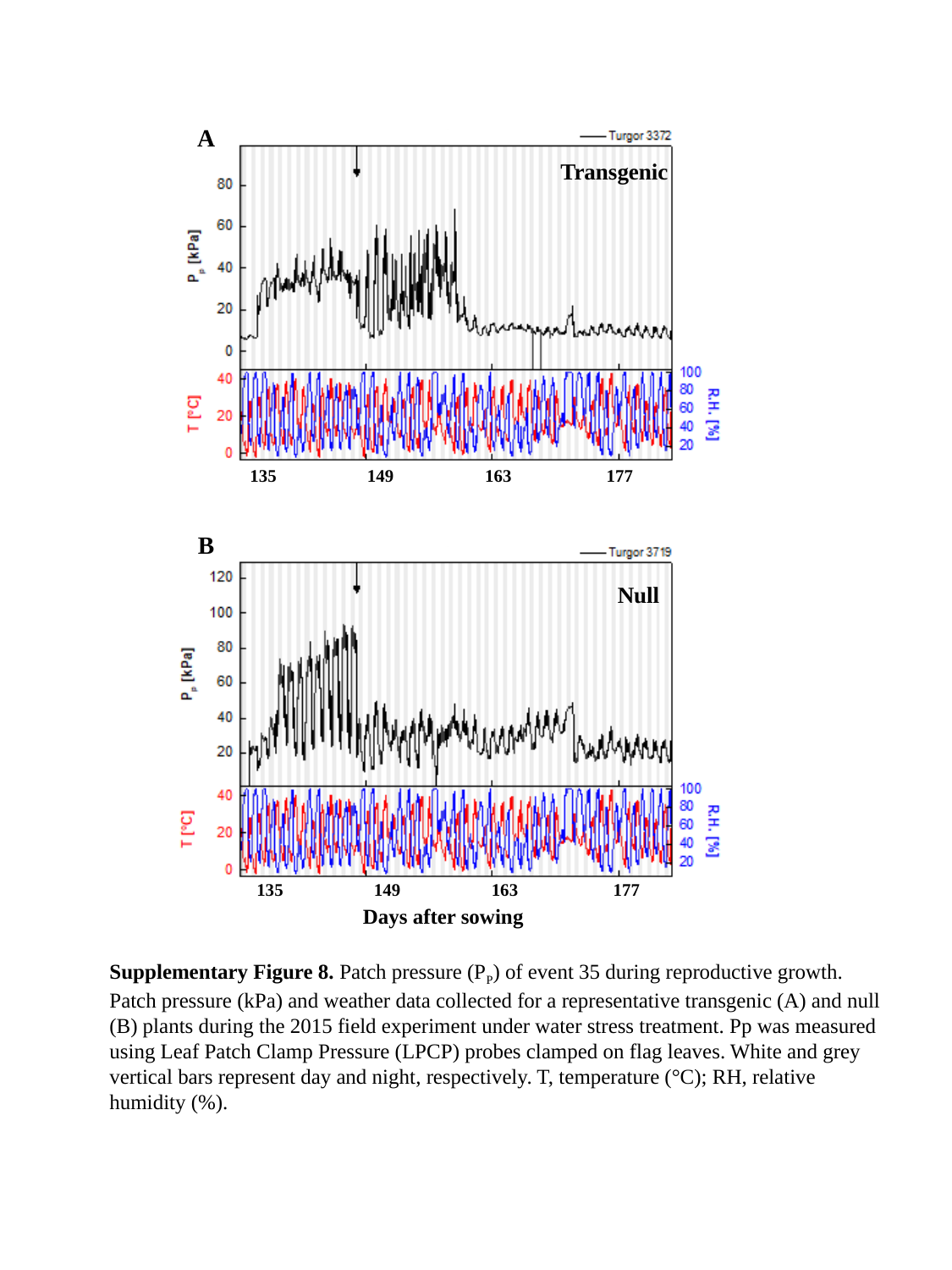

A
Transgenic
163
177
135
149
B
Null
163
177
135
149
Days after sowing
Supplementary Figure 8. Patch pressure (PP) of event 35 during reproductive growth. Patch pressure (kPa) and weather data collected for a representative transgenic (A) and null (B) plants during the 2015 field experiment under water stress treatment. Pp was measured using Leaf Patch Clamp Pressure (LPCP) probes clamped on flag leaves. White and grey vertical bars represent day and night, respectively. T, temperature (°C); RH, relative humidity (%).

## Slide 9
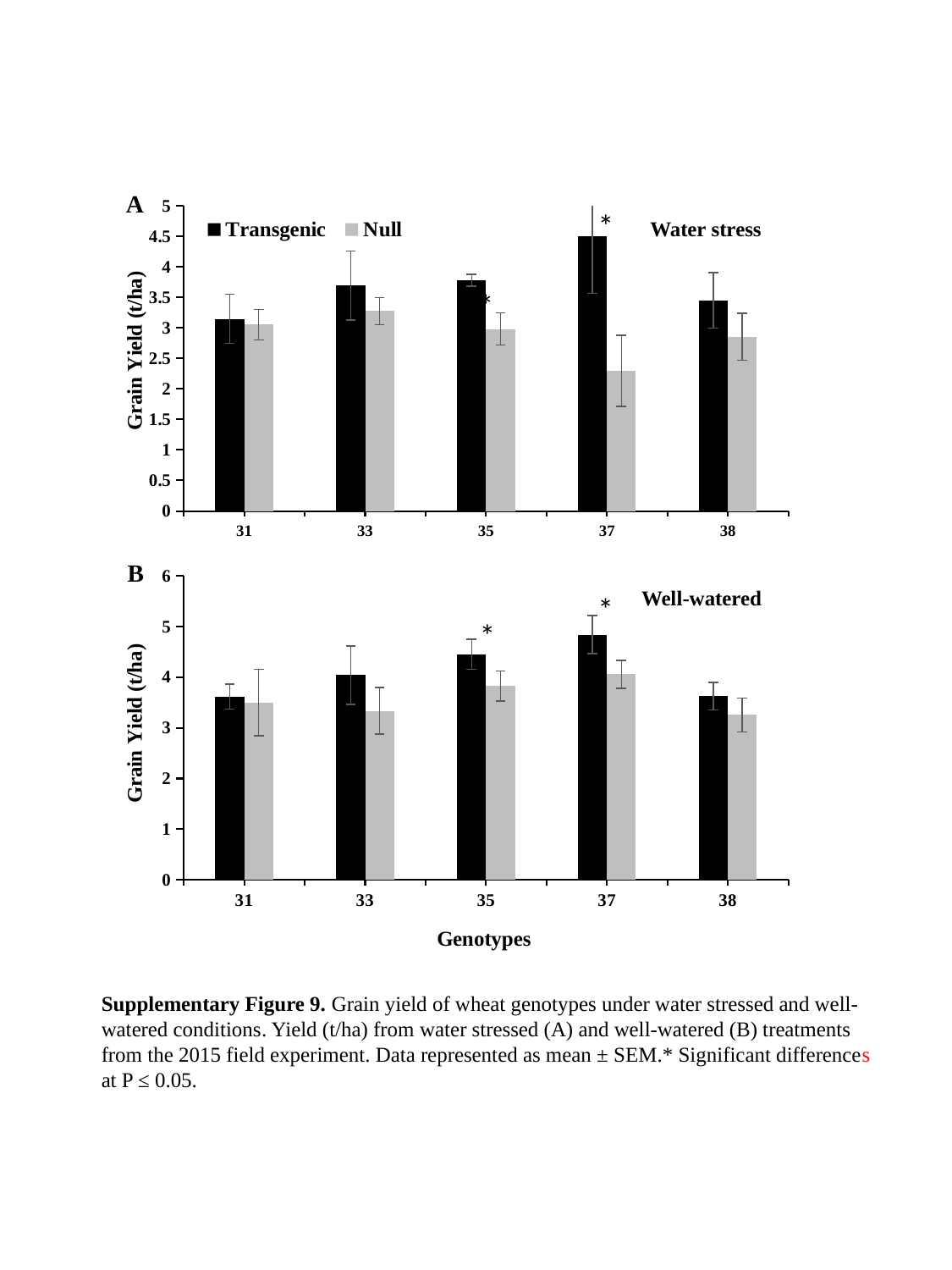

### Chart
| Category | Transgenic | Null |
|---|---|---|
| 31 | 3.1494583333333335 | 3.0533333333333332 |
| 33 | 3.6942777777777778 | 3.27525 |
| 35 | 3.7784444444444447 | 2.9805833333333336 |
| 37 | 4.506777777777778 | 2.292666666666667 |
| 38 | 3.4516666666666667 | 2.8521666666666663 |A
*
Water stress
*
B
### Chart
| Category | Transgenic | Null |
|---|---|---|
| 31 | 3.613333333333335 | 3.500055555555557 |
| 33 | 4.0377777777779 | 3.3333333333333335 |
| 35 | 4.4512222222222135 | 3.8217222222222236 |
| 37 | 4.837555555555554 | 4.056388888888891 |
| 38 | 3.6253333333333346 | 3.2531666666666696 |Well-watered
*
*
Supplementary Figure 9. Grain yield of wheat genotypes under water stressed and well-watered conditions. Yield (t/ha) from water stressed (A) and well-watered (B) treatments from the 2015 field experiment. Data represented as mean ± SEM.* Significant differences at P ≤ 0.05.
